# Natural fibers in sustainable materials: extraction technologies, fiber modification, and performance–sustainability relationships

**DOI:** 10.1039/d5ra09029f

**Published:** 2026-02-20

**Authors:** Sayam

**Affiliations:** a Department of Fabric Engineering, Barishal Textile Engineering College Barishal 8200 Bangladesh mahbub.sayamm@gmail.com

## Abstract

Natural fibers have emerged as sustainable alternatives to synthetic materials in textile applications due to their biodegradability, renewability, and diverse functional properties. Derived from plant, animal, and mineral sources, fibers such as cotton, jute, hemp, flax, silk, and bamboo demonstrate excellent physical and mechanical characteristics suitable for apparel, home furnishing, and industrial textiles. Their utilization supports circular production systems, reduces dependency on petroleum-based resources, and minimizes environmental impacts such as pollution and greenhouse gas emissions. The review comprehensively analyzes the properties, extraction methods, and modifications of natural fibers, highlighting advanced processing techniques like enzymatic and microwave-assisted retting that enhance quality while lowering ecological footprints. Furthermore, it discusses the socio-economic benefits of natural fiber industries, including rural employment generation and resource-efficient production from agricultural residues. Despite challenges related to variability, water use, and processing costs, ongoing research in fiber surface treatment and composite technology is driving innovation and improving performance. The integration of natural fibers into textile manufacturing represents a crucial pathway toward achieving sustainable fashion, environmental stewardship, and a circular bio-economy.

## Abstract 2

Natural fibers have emerged as sustainable alternatives to synthetic materials in textile applications due to their biodegradability, renewability, and diverse functional properties.

## Graphical abstract

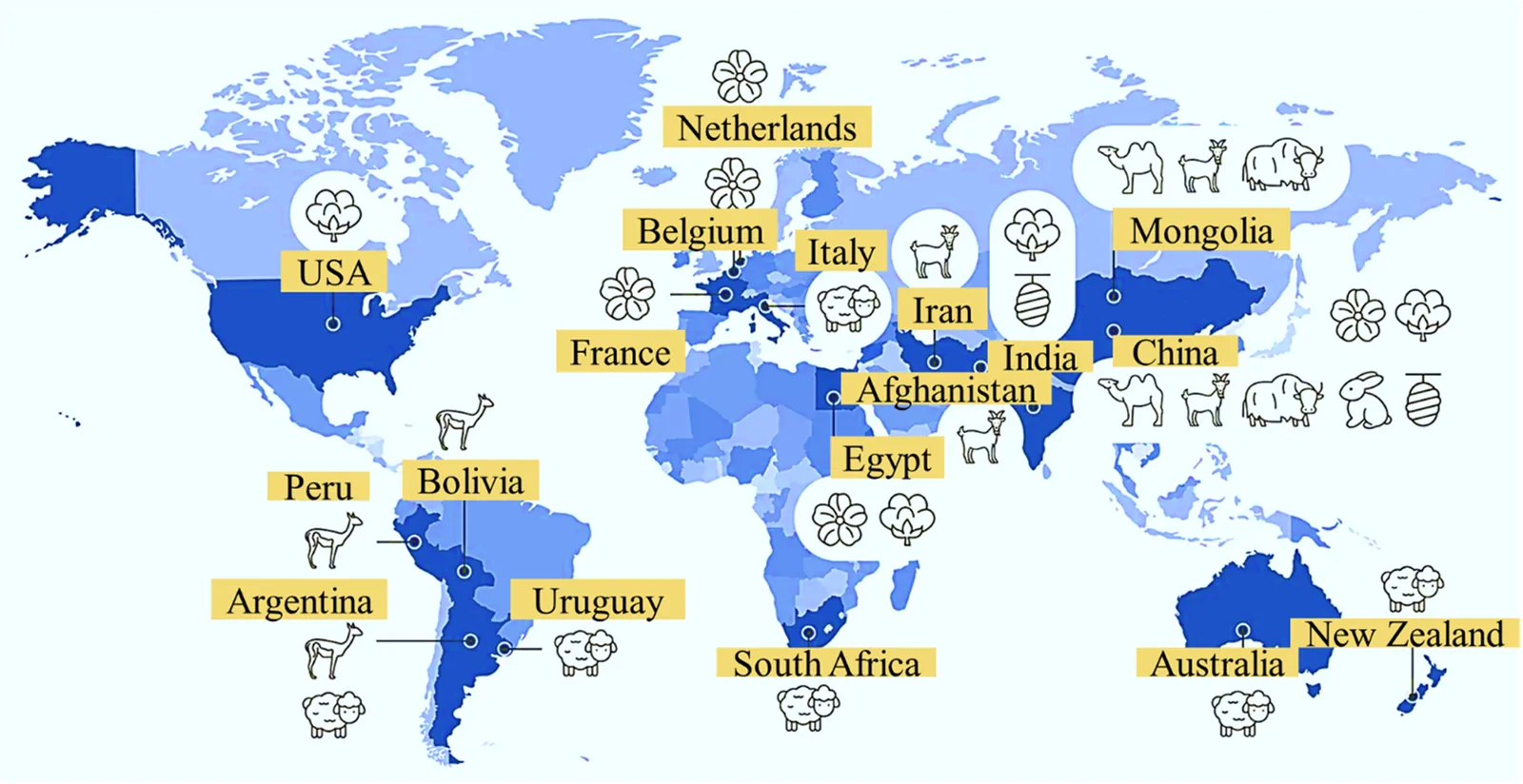

## 1 Introduction

Material selection plays a critical role in sustainable engineering design, as the physical and mechanical properties of materials determine product performance, cost, manufacturability, and environmental impact. Among eco-friendly fibers, natural fibers have gained wide attention due to their ease of processing, high productivity, and potential for cost reduction.^1,2^ Their properties can be optimized by varying the type and proportion of reinforcement and matrix phases.^3^ In this context, natural fibers have emerged as promising reinforcements in polymer composites because they are renewable, biodegradable, low-cost, and widely available.^4,5^ The density of natural fibers (1.2–1.6 g cm^−3^) is significantly lower than that of glass fiber (2.4 g cm^−3^), which enables the fabrication of lightweight composites with good specific mechanical properties. Consequently, natural fibers such as hemp, jute, sisal, banana, coir, and kenaf have found applications in various sectors, including automotive interiors, furniture, construction, packaging, and shipping pallets.^6^ Their utilization also aligns with the growing demand for environmentally friendly and sustainable materials in textile and composite manufacturing.

Natural fibers are obtained from different biological sources, including plants and animals, such as wool, chicken feathers, hair, and silk.^7^ Plant-based fibers consist mainly of cellulose, hemicellulose, pectin, lignin, waxes, and other extractives that define their structural, chemical, and mechanical characteristics. However, their hydrophilic nature often limits compatibility with hydrophobic polymer matrices, leading to poor interfacial adhesion and moisture sensitivity. Chemical treatments such as alkali, silane, or acetylation are commonly used to reduce hydroxyl groups, increase surface roughness, and improve fiber–matrix bonding.^8,9^ Meanwhile, the rising global emphasis on sustainability, depletion of petroleum-based resources, and stricter environmental regulations have accelerated interest in bio-based materials.^10^ Agricultural residues such as cereal straw, corn stalks, cotton residues, bagasse, and grasses offer abundant and low-cost lignocellulosic biomass. The effective utilization of these residues can minimize open-field burning, reduce pollution, and promote rural industrialization and circular economy models.^11^ These bioresources thus play a dual role by addressing waste management issues and providing a renewable feedstock for textile industries.

The physical and chemical characteristics of natural fibers, such as cell-wall thickness, fiber dimensions, cross-sectional geometry, and lumen structure, along with the relative composition of cellulose, hemicellulose, and lignin, significantly influence their tensile strength, stiffness, thermal stability, moisture absorption, and biodegradation behavior.^12^ Moreover, the availability and characteristics of these fibers vary across geographical regions due to differences in climate, soil conditions, and agricultural practices. Understanding the global distribution of bioresources is therefore essential to identify region-specific fibers for sustainable industrial applications. In this context, the next section of this paper focuses on the bioresources and geographical distribution of natural fibers across different countries, providing an overview supported by a global map representation. The objective of this review is to systematically compile and compare plant-, animal-, and mineral-based natural fibers with respect to their origin, classification, key physical, chemical, and thermal properties, and modification routes relevant to textile and composite processing. Particular attention is given to linking fiber structure and surface treatments with energy demand during processing and the resulting environmental and sustainability implications. By organizing dispersed data from existing studies into a unified framework that connects material properties, application-based classification, and sustainability assessment, this review provides a clear reference for selecting natural fibers for textile and composite applications based on functional and environmental criteria.

## 2 Global distribution of natural fibers

**Fig. 1:**
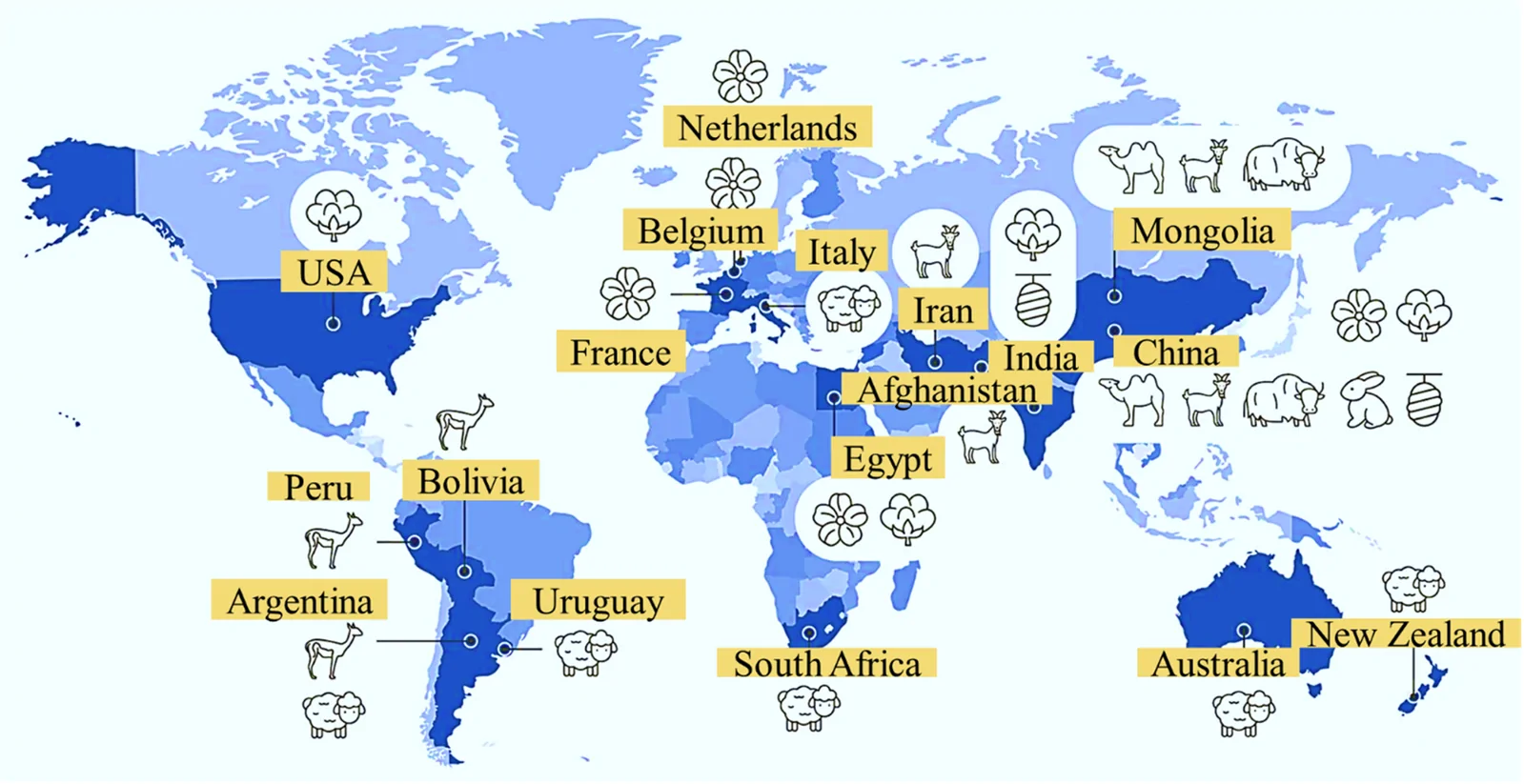
Global distribution of natural fiber sources and their major production regions.

Natural fibers are abundantly available worldwide and are derived primarily from plant, animal, and mineral sources. Fig. 1 illustrates the global distribution of natural fiber availability by country, while Table 1 presents production statistics of major natural fibers. Plant-based fibers dominate global production due to their wide availability, renewability, and adaptability to tropical and subtropical climates.

**Table 1:** Worldwide production of natural fibers with corresponding producing countries

Country name	Production (tons)	Source	Ref.
Bangladesh, China, India	2 300 000	Jute	13–15
Bangladesh, China, India, Indonesia, Malaysia	160 000 000	Rice husk	16 and 17
Bangladesh, China, India, Indonesia, Malaysia	579 000	Rice straw	15 and 18
Bangladesh, India, United States	970 000	Kenaf	13–15 and 19
Belgium, Canada, France	830 000	Flax	13–15 and 19
Brazil, China, India	75 000 000	Sugarcane bagasse	13–15 and 19
Brazil, Kenya, Tanzania	378 000	Sisal	13–15 and 19–22
Brazil, China, India, Philippines	100 000	Ramie	13–15 and 19–22
Canada, China, United States	1 750 000 000	Wood fiber	14 and 18
China, France, Philippines	214 000	Hemp	23 and 24
China, India, Indonesia, Malaysia, Philippines	30 000 000	Bamboo	13–15 and 19
Costa Rica, Ecuador, Philippines	70 000	Abaca	13–15, 19 and 22
India, Malaysia, Philippines, Sri Lanka	100 000	Coir	25
Indonesia, Malaysia	40 000	Oil palm	26
Indonesia, Philippines, Thailand	74 000	Pineapple leaf	13–15 and 19–22
Australia, China, New Zealand, United Kingdom, Argentina	1 100 000	Wool	27 and 28
China, India	91 221	Silk	29 and 30
China, Mongolia	25 000	Cashmere	31
South Africa	5000	Mohair	31
Peru, Bolivia, Chile	7000	Alpaca	31
Peru	<10	Vicuña	31
Russia, China, Brazil, Kazakhstan	2 000 000	Asbestos	32

Musa plants (*Musa acuminata*), belonging to the Musaceae family, are native to Southeast Asia and generate high-fiber biomass from bunches, pseudostems, leaves, and stalks.^33^ Banana fiber is widely available in tropical regions such as Malaysia and South India and is the fourth most important crop in developing countries.^34^ Abaca (*Musa textilis*) is mainly cultivated in Talaud, North Sulawesi, where five superior local varieties were released in 2019.^35^ Pineapple leaf fiber (*Ananas comosus* L. Merr.) is an underutilized agricultural waste material abundant in Southeast Asia, India, and South America.^36^ Indonesia contributes approximately 23% of global pineapple production, supported by favorable tropical climatic conditions.

Bamboo is a lignocellulosic fiber from the Graminae family with broad application potential. Bamboo fibers are aligned longitudinally and exhibit good mechanical performance.^37^ After China and India, Indonesia ranks third in bamboo production and hosts around 160 bamboo species, including 88 endemic species. Betung bamboo (*Dendrocalamus asper*) is among the most commonly utilized species.^38^ Cotton fiber (*Gossypium* sp.), a fruit fiber widely used in textiles and healthcare products, is mainly produced in the USA, Egypt, India, and Brazil. Cotton fibers are classified into long, medium, and short categories based on fiber length.^39^ Despite increasing demand, cotton production remains limited in some regions due to low productivity and reduced farmer interest.

Ramie (*Boehmeria nivea*) is a fast-growing bast fiber known for its compatibility with other fibers and high productivity. Global ramie production is approximately 100 thousand tons per year, exceeding abaca production at around 70 thousand tons per year.^40–42^ Sisal (*Agave sisalana*) is a drought-resistant leaf fiber plant that can be cultivated on marginal land and contributes nearly half of global hard fiber textile production.^43^ Indonesia produces about 500 tons of sisal fiber annually, extracted from plant leaves.^42^ Kenaf (*Hibiscus cannabinus* L.) is adaptable to diverse soil conditions, with productivity ranging from 2.0 to 4.0 tons of dry fiber per hectare, and is mainly produced in India and Pakistan.^44^ Corn plants, widely cultivated across Asia, also offer high potential as natural fiber sources from stems, leaves, and husks.

Animal-based natural fibers contribute smaller volumes but higher economic value compared to plant fibers. Wool is the most significant animal fiber and plays an important role in the economies of producing countries.^27^ Major wool-producing regions include Australia, China, New Zealand, the United Kingdom, and Argentina, with Australia contributing approximately 25% of global greasy wool exports by value.^27^ Wool prices generally range from USD 3 to 12 per kilogram, depending on micron count and origin.^28^ Silk production, although limited in volume, holds high economic value. Global raw silk production reached 91 221 metric tons between 2011 and 2022, with China and India dominating production and China maintaining a historically strong position in processing.^29,30^ Raw silk typically commands prices between USD 30 and 60 per kilogram.^29^

Luxury animal fibers such as cashmere, mohair, alpaca, vicuña, camel hair, and angora are characterized by low production volumes and exceptionally high market value.^45^ China and Mongolia dominate global cashmere production, with China supplying 60–70% of total output.^31^ South Africa contributes approximately 50% of global mohair production, while alpaca fiber production is concentrated in Peru, accounting for over 75% of global supply.^31^ Although vicuña fiber is produced in negligible quantities, it is among the most valuable natural fibers, with prices estimated at USD 1500–3000 per kilogram.^31^ Economically, East Asia leads global animal fiber production value, followed by Western Europe and Oceania for fine wool, and Andean countries for specialty fibers.^28,31^ Increasing consumer preference for high-quality and sustainable textiles has further strengthened demand for animal fibers.^28^ Keratin-based animal fibers are also being explored for sustainable composite applications due to their flame-retardant behavior and reinforcement potential.^46^

Mineral-based fibers have historically contributed to natural fiber use, with asbestos being the most prominent due to its high tensile strength, chemical resistance, and thermal stability.^47^ However, asbestos is classified as a human carcinogen and is banned or strictly regulated in more than 70 countries.^48^ Despite these restrictions, global asbestos production remained approximately 2 million tons per year as of 2016, with about 90% originating from Russia, China, Brazil, and Kazakhstan.^32^ Although global consumption has declined, asbestos use increased in several developing regions as of 2013, particularly in the Asia-Pacific, which consumed over half of global asbestos production in 2011.^49,50^ Due to the long latency period of asbestos-related diseases, ranging from 20 to 50 years, health impacts persist even after complete bans, with an estimated 125 million people occupationally exposed worldwide and approximately 255 000 deaths annually attributed to asbestos-related causes.^51^

The decline of asbestos has increased interest in alternative mineral fibers for composite applications. While mineral fibers differ from biologically derived natural fibers, they are often discussed alongside them in engineering contexts.^52^ Basalt fiber, derived from volcanic rock, exhibits favorable mechanical performance, thermal stability, chemical durability, moisture resistance, and recyclability. Production is concentrated in Russia, China, India, and Germany, although global output remains limited and detailed production statistics are not publicly consolidated.^53^

## 3 Properties of natural fibers

### 3.1 Physical and mechanical properties

Natural fibers exhibit a wide range of physical and mechanical properties depending on their botanical source, microstructure, and chemical composition. In addition to plant-based fibers, animal- and mineral-based natural fibers exhibit distinct physical and mechanical behaviors due to their protein-based and inorganic compositions, respectively. Table 2 shows the physical and mechanical properties of various natural fibers collected from different literature sources. The density of these fibers typically ranges between 0.55 and 1.60 g cm^−3^, which is significantly lower than that of synthetic fibers, making them attractive for lightweight composite applications. Animal fibers such as wool and silk also exhibit relatively low densities and favorable strength-to-weight ratios, whereas mineral fibers generally show higher density and lower elongation characteristics.^54^

**Table 2:** Physical and mechanical properties of different natural fibers

Natural fiber	Density (g cm^−3^)	Diameter (µm)	Tensile strength (MPa)	Young's modulus (GPa)	Elongation at break (%)	Ref.
Abaca	0.83	1.5	900	12–13.8	3–12	55
Agave	1.20	126–344	—	—	7.07	56
Bamboo	0.51–0.72	21–26	225	17.2	3	57
Banana	1.35	50–250	600	17.85	3.36	56
Coir	1.15	100–450	131–175	4–6	4–6	58 and 59
Cotton	1.51–1.6	2–7	287–597	5.5–12.6	3–10	58 and 60
Date	0.99	—	309	11.32	2.73	56
Flax agatha	1.50–1.53	14.4–15.6	962–1800	46–96	—	61
Henequen	1.4	—	430–580	—	—	14
Hemp	1.48	26.5	514	24.8	1.5–4	61–63
Hardwood	0.3–1.2	—	—	5.2–37.9	—	64 and 65
Jute	1.3–1.45	25–200	393–773	13–26.5	1.5–1.8	20 and 58
Kenaf	1.3	—	233	40–53	1.8	66–68
Kudzu	—	—	130–418	—	—	64
Luffa	0.82	25–60	385	12.2	—	66
Napier	0.36	—	73	5.68	1.4	69
Nettle	0.72	15.5–24.3	1594	59–115	—	70 and 71
Okra	—	61–93	184–557.3	8.9–11.8	4–8	56
Oil palm	0.7–1.55	—	—	1–9	8–25	64
Palmyrah	1.09	8	180–215	4.4–6.1	—	72
Pineapple leaf	1.07–1.50	20–80	413–1627	34.5–82.5	1.6	66
Ramie	1.51	34	400–968	60–128	3.6–3.8	58 and 61
Rice husk	2.30–2.36	45	—	—	—	73
Saw dust	0.31–0.32	75–600	16–24	(0.2–0.36) × 10^−3^	—	74 and 75
Sea grass	1.50	5	453–692	3.1–3.7	13–26.3	56
Sisal	1.45	50–200	468–640	9.4–22	3–6	20 and 58
Softwood	1.5	—	1000	0.04	4.4	55
Sugar palm	1.29	50–800	190.29	3.69	—	72
Sugarcane bagasse	0.33	67–312	222	27.1	—	66
Wood chips	0.28–0.32	3000–16000	—	(0.25–0.33) × 10^−3^	—	75
Alpaca wool	—	18–35	—	—	25–35	76
Sheep wool	—	22–55	100–200	—	25–35	76
Goat wool	—	10–35	—	—	25–35	76
*Bombyx mori* silk	—	10–24	350–500	5–12	10–24	77
Tussah silk	—	50–70	400–600	9–15	10–25	77
Asbestos	—	—	200–1000	—	<3	78

Abaca fiber generally possesses a density of about 1.50 g cm^−3^, showing elongation between 3 and 12%, tensile strength reaching up to 980 MPa, and a modulus of elasticity around 12–72 GPa, indicating high stiffness and strength as reported by Biagiotti *et al.*^79^ Bagasse fiber, on the other hand, shows much lower density, ranging from 0.55 to 1.25 g cm^−3^, with tensile strength values varying from 20 to 350 MPa and modulus between 0.5 and 27 GPa. The relatively lower strength of bagasse compared to abaca is attributed to its higher lignin content and porous cell structure as indicated by Yan *et al.*^80^

Bamboo fiber demonstrates an intermediate behavior, with density values between 0.60 and 1.50 g cm^−3^, elongation varying from 1.3 to 7%, tensile strength in the range of 140–800 MPa, and modulus between 11 and 36 GPa. These values suggest that bamboo fiber offers a favorable balance between flexibility and strength, a property emphasized in the works of Sathishkumar *et al.*^56^ and Sujaritjun *et al.*^81^ Similarly, banana fiber exhibits densities between 0.75 and 1.35 g cm^−3^, elongation at break ranging from 1–9%, tensile strength from 54–914 MPa, and modulus between 7.7 and 32 GPa. The moderate stiffness and strength of banana fiber are linked to its semicrystalline cellulose regions and the presence of natural microvoids.^82^

Coconut fiber, being highly lignified, exhibits a comparatively low density ranging from 0.38 to 1.10 g cm^−3^, tensile strength between 83 and 222 MPa, and modulus values close to 12–32 GPa. Despite the low tensile properties, its high elongation makes it suitable for energy-absorbing and cushioning applications, as highlighted by Danso *et al.*^83^ Coir fiber, another lignocellulosic fiber from the same family, possesses densities around 1.15–1.25 g cm^−3^, an extremely high elongation of 15–51%, tensile strength from 95 to 304 MPa, and modulus values up to 6 GPa. The high extensibility and ductile nature of coir fiber are consequences of its elevated lignin content.^84,85^

Cotton fiber is comparatively dense, around 1.50–1.60 g cm^−3^, with elongation between 3 and 10%, tensile strength ranging from 200 to 800 MPa, and modulus values within 5.5–13 GPa. Its moderate stiffness and consistent strength have been widely reported in studies by Wambua *et al.*^86^ and Saba *et al.*^87^ Flax fiber, one of the strongest bast fibers, shows a density near 1.50 g cm^−3^, tensile strength from 345 to 2000 MPa, elongation between 1 and 4%, and modulus as high as 103 GPa. The superior strength and stiffness of flax are associated with its high cellulose content and low microfibrillar angle.^88,89^

Zhang *et al.*^90^ and Pickering *et al.*^91^ demonstrated that hemp fiber shows similar performance, with density between 1.40 and 1.50 g cm^−3^, tensile strength in the range of 270–1110 MPa, elongation from 1 to 4%, and modulus values up to 90 GPa. The high mechanical properties of hemp are the result of its high cellulose crystallinity and strong fibrillar alignment. Jute fiber, another commonly used bast fiber, exhibits density values from 1.30 to 1.50 g cm^−3^, elongation between 1.5 and 3%, tensile strength of 200–800 MPa, and modulus between 10 and 55 GPa. The variability in jute's tensile strength is due to its nonuniform fibrillar structure and variations in retting quality.^92,93^

Pineapple leaf fiber possesses excellent mechanical characteristics with density between 1.07 and 1.56 g cm^−3^, elongation of up to 14.5%, tensile strength reaching 1627 MPa, and modulus between 60 and 82 GPa. These outstanding values are mainly due to its high cellulose content and strong inter-fibrillar bonding, as observed by Abiola *et al.*^82^ and Saba *et al.*^87^ Ramie fiber also shows exceptional stiffness, with density around 1.50 g cm^−3^, elongation between 1.2 and 4%, tensile strength from 400 to 938 MPa, and modulus as high as 128 GPa. The strong crystalline regions and highly oriented microfibrils of ramie account for its excellent load-bearing capacity.^19,85^

Sisal fiber demonstrates a moderate to high level of mechanical strength, with density between 1.30 and 1.50 g cm^−3^, elongation from 2 to 7%, tensile strength varying between 350 and 855 MPa, and modulus between 9 and 38 GPa. The good balance of stiffness and ductility in sisal is largely due to its moderate cellulose and lignin contents.^93–95^

Animal fibers such as wool and silk are primarily protein-based materials. Wool fibers consist mainly of keratin with a helical molecular structure and disulfide bonds, which contribute to their elasticity and resilience.^76^ This structure results in moderate tensile strength, typically around 100–200 MPa, and high elongation at break in the range of 25–35%, as well as good thermal insulation and moisture absorption properties.^96^ Wool fibers have also been investigated as reinforcements in polymer composites, showing improved mechanical performance.^9,10^ Silk fibers, composed mainly of fibroin with highly ordered β-sheet structures, exhibit higher tensile strength and stiffness compared to wool, and their mechanical properties can be further enhanced through processing and modification, reaching strengths comparable to spider silk.^77^

In contrast, mineral fibers mainly refer to asbestos, which consists of naturally occurring fibrous silicate minerals. Asbestos fibers exhibit high tensile strength, typically ranging from 200 to 1000 MPa, combined with very low elongation at break, usually below 3%, as well as excellent thermal, chemical, and electrical resistance. Although these properties historically made asbestos valuable in industrial applications, its use has been largely discontinued due to its carcinogenic nature and associated health risks.^78^

In general, fibers such as flax, hemp, ramie, pineapple, and sisal exhibit the highest tensile strength and modulus values, confirming their suitability for high-performance structural composites. In contrast, fibers like coir, bagasse, and coconut, while possessing lower tensile strengths, display higher elongations and energy absorption capabilities, making them better suited for flexible or impact-resistant materials. Animal fibers are particularly suitable for insulation, damping, and bio-composite applications due to their renewability and biodegradability, whereas mineral fibers are characterized by extreme durability but limited applicability because of environmental and health concerns.

### 3.2 Biological properties

One of the most significant biological properties of natural fibers is their biodegradability.^97^ Unlike many synthetic plastics that persist in the environment for centuries, natural fibers can be decomposed by microorganisms (*e.g.*, bacteria, fungi) into simpler compounds, such as carbon dioxide, methane, water, and biomass.^98^ This characteristic makes them a sustainable alternative, reducing waste accumulation and environmental pollution. The rate and extent of biodegradation are influenced by the fiber's physicochemical structure, including its chemical composition and crystallinity, as well as environmental conditions like temperature, moisture, and microbial activity, as shown in Table 3.^99^ Cellulose, a primary component of plant-based natural fibers, is generally biodegradable. The degradation process is often assessed through weight loss, strength reduction, and morphological changes over time. For instance, wool, a natural keratin fiber, and cotton, a natural cellulose fiber, exhibit significant biodegradability in natural soil and aqueous media at 35 °C over 42 days.^100^ Similarly, researchers have investigated the degradation of cellulose fibers functionalized with nanoparticles in soil burial tests and model compost, demonstrating their potential for sustainable disposal after use.^101^ The presence of lignin, another major component in lignocellulosic fibers, can impact biodegradability by providing rigidity and resistance to microbial degradation.^102^ However, biochar derived from lignocellulosic biomass, such as walnut shells, can enhance microbial communities' abundance and degradation of lignocellulosic materials in anaerobic digestion.^103^ Cellulose, with its linear chain of glucose monomers, provides strength, while the more branched hemicellulose and complex phenolic lignin contribute to the fiber's overall structure and resistance.^102^

**Table 3:** Comparative biological characteristics of natural plant-based and animal-based fibers

Property	Plant-based natural fibers	Animal-based natural fibers
Biodegradation behavior	Rapidly biodegradable in soil and aquatic environments *via* microbial activity.^104,105^ Degradation rate is governed by polysaccharide accessibility and inversely correlated with lignin content^106^	Biodegradable under natural and composting conditions through microbial enzymatic activity targeting protein backbones^104,105^
Biodegradation mechanism	Enzymatic hydrolysis by cellulases and hemicellulases, leading to progressive mass and mechanical property loss^107,108^	Proteolytic degradation of keratin and fibroin chains by microorganisms^104,105^
Representative evidence	Hemp, jute, sisal, ramie, regenerated cellulose, and *Tipuana tipu* fruit fibers exhibit significant degradation, with compositional dependence (cellulose–hemicellulose–lignin ratio) influencing biodegradation kinetics^106,108^	Wool demonstrates substantial biodegradation in soil, aqueous, and aerobic composting environments, comparable to or exceeding that of PLA and cotton^104,105^
Cytocompatibility	Generally high cytocompatibility owing to chemical inertness and absence of toxic degradation products; suitability for biomedical use can be further enhanced through surface modification^109^	Excellent cytocompatibility and bioactivity, particularly for silk fibroin and keratin, supporting extensive application in tissue engineering and regenerative medicine^110^
Antimicrobial potential	Limited intrinsic antimicrobial activity; performance is highly tunable through chemical functionalization or incorporation of antimicrobial agents^109^	Typically lacks strong inherent antimicrobial activity; functional modification is required to impart antimicrobial behavior^110^
Additional biological functions	Effective biosorbents for metal ions due to abundant hydroxyl and carboxyl functional groups; also used as bio-reinforcement in microbially enhanced self-healing composites^110^	Suitable for engineered bio-composites with tailored biological and mechanical properties; allergenic responses depend largely on fiber morphology and processing rather than intrinsic chemistry^110^

### 3.3 Chemical properties

The chemical composition of natural fibers largely determines their physical, mechanical, and interfacial behavior in textile and composite applications. Natural fibers are complex biopolymers composed primarily of cellulose, hemicellulose, lignin, pectin, waxes, and minor extractives, as shown in Table 4, with their relative proportions varying according to the botanical source, growth conditions, and extraction method.^111^ Cellulose, a linear polysaccharide of β-d-glucopyranose units, forms the main structural framework and contributes to high tensile strength, stiffness, and dimensional stability through extensive intra- and intermolecular hydrogen bonding.^112^ The degree of polymerization and crystallinity of cellulose significantly affect the mechanical and sorption behavior of fibers.^113^ Hemicellulose, an amorphous matrix of short-chain polysaccharides, binds microfibrils together and influences flexibility and hydrophilicity, although its low thermal and biological stability makes fibers more susceptible to moisture absorption and microbial degradation.^114^ Lignin, a three-dimensional phenolic polymer, acts as a cementing material that provides rigidity, ultraviolet resistance, and protection against microbial attack; however, high lignin content generally reduces flexibility and spinnability.^115,116^ Pectin and surface waxes determine fiber smoothness and dyeability, and their partial removal during retting or alkali treatment improves adhesion and wettability in subsequent processing.^117^

**Table 4:** Chemical composition of natural fibers

Fiber	Hemicellulose (wt%)	Cellulose (wt%)	Lignin (wt%)	Ref.
Bamboo	30	26–43	21–31	118
Coir	0.15–0.25	32–43	40–45	119
Date palm	18–25	41–46	20–27	120
Banana	38.54	43.46	9	121
Bagasse	16.8	55.2	25.3	122
Abaca	20–25	56–63	7–9	123
Jute	14–20	61–71	12–13	124
Sisal	—	65	9.9	122
Hemp	15	68	10	125
Ramie	13–16	68.6–76.2	0.6–0.7	126
Flax	18.6–20.6	71	2.2	126
Kenaf	20.3	72	9	120
Pineapple	—	81	12.7	118
Cotton	5.7	82.7–90	<2	124

Typical compositional ranges reported in literature indicate that cotton contains about 88–96% cellulose with minimal lignin (<1%), jute and hemp contain 60–70% cellulose, 15–22% hemicellulose, and 8–12% lignin, while coir is distinguished by a low cellulose fraction (32–43%) but exceptionally high lignin content (40–45%), conferring remarkable durability and water resistance.^125^ Recent studies further demonstrate variability within plant fibers; for example, *Ficus racemosa* fibers contain 72.36 wt% cellulose, 11.21 wt% hemicellulose, and 10.45 wt% lignin, while *Acacia arabica* fibers exhibit 68.61 wt% cellulose, 15.63 wt% hemicellulose, and 9.47 wt% lignin.^127,128^ Similarly, *Ficus carica* bark fibers show 62.1 wt% cellulose, 12.3 wt% hemicellulose, and 9.6 wt% lignin, confirming that differences in botanical origin significantly influence chemical composition and, consequently, fiber performance.^129^ The density of these fibers also reflects compositional variation, with *Ficus racemosa* fibers exhibiting a density of approximately 895 kg m^−3^.^127^ Protein-based fibers such as wool and silk differ fundamentally, being composed almost entirely of keratin and fibroin proteins, respectively, whose peptide linkages and sulfur cross-bridges dictate their elasticity and resistance to deformation.^130^

The abundance of hydroxyl groups in cellulose and hemicellulose renders most plant fibers chemically reactive, enabling surface modification through alkali treatment, silane coupling, acetylation, or grafting reactions that enhance compatibility with hydrophobic polymer matrices. While such treatments improve adhesion and reduce moisture affinity, the fibers remain sensitive to strong acids and oxidizing agents that can cleave glycosidic bonds and reduce molecular weight.^131^ These compositional differences clearly distinguish plant fibers rich in polysaccharides from protein-based animal fibers and inorganic mineral fibers, which lack reactive hydroxyl groups and therefore require different modification strategies or are unsuitable for chemical functionalization in composite systems. The chemical characteristics of natural fibers govern their intrinsic performance, such as strength, durability, and dyeability, and also dictate their processing behavior and environmental stability, making chemical composition a critical factor in designing fibers for advanced textile and composite applications.^132^

### 3.4 Thermal properties

The thermal properties of natural fibers are critical in determining their processing behavior, dimensional stability, and suitability for textile and composite applications. In general, cellulosic fibers such as cotton, flax, jute, hemp, and sisal display limited thermal stability, with degradation typically beginning around 220–250 °C, corresponding to the breakdown of hemicellulose and amorphous cellulose regions, followed by major mass loss between 300–350 °C due to cellulose decomposition.^133^ The residual char formation above 400 °C is mainly attributed to lignin, which decomposes over a broader temperature range (200–500 °C) because of its aromatic, cross-linked polymeric structure.^134,135^ The higher the lignin content, as in coir fiber, the greater the char yield and thermal resistance, which enhances its performance in thermal insulation and composite reinforcement applications. Conversely, low-lignin fibers like cotton tend to ignite easily and produce minimal char residue. In contrast, mineral-based fibers exhibit superior thermal stability compared to plant- and animal-derived fibers, as organic constituents such as cellulose, hemicellulose, lignin, and proteins typically degrade between 200 and 300 °C, whereas mineral fibers can withstand temperatures exceeding 500 °C due to their crystalline silicate structures and strong ionic or covalent bonding.^136,137^

Thermal conductivity and heat capacity of natural fibers are generally low, ranging between 0.04 and 0.06 W m^−1^ K^−1^, making them excellent thermal insulators for protective clothing and building textiles.^138^ Moisture content plays a significant role, as absorbed water acts as a heat sink and influences the specific heat capacity and thermal diffusivity of fibers. The crystalline regions of cellulose resist thermal motion, while amorphous regions soften earlier, leading to shrinkage and loss of mechanical integrity under high temperatures.^139^ For mineral-based fibers, thermal conductivity is influenced by parameters such as density, fiber diameter, and operating temperature. Basalt fiber insulation materials, for example, exhibit thermal conductivity values ranging from approximately 0.038 to 0.053 W m^−1^ K^−1^ at mean temperatures between 100 °C and 400 °C, with temperature having a more pronounced effect than density.^140,141^ Basalt fiber insulation with densities of 50 and 150 kg m^−3^ shows only minor differences in thermal conductivity at equivalent temperatures, indicating that heat transfer behavior is primarily temperature-dependent.^140^ The inherently porous and fibrous structure of mineral fibers contributes significantly to their low thermal conductivity by trapping air within the material, which impedes heat transfer through conduction, convection, and radiation. In such porous insulation systems, total thermal conductivity is governed by the combined contributions of solid conduction, convective heat transfer, and radiative transfer (*k* = *k*_c_ + *k*_conv_. + *k*_r_).^142^

In the case of protein-based fibers, silk exhibits thermal degradation around 280–290 °C due to peptide bond cleavage in fibroin,^143^ while wool begins to degrade near 200 °C as disulfide linkages in keratin are disrupted, releasing sulfur-containing volatiles.^144^ This comparatively lower thermal stability limits the use of animal fibers in high-temperature environments but supports their application in thermal insulation and comfort-related textiles. Thermal analysis techniques such as Thermogravimetric Analysis (TGA) and Differential Scanning Calorimetry (DSC) are widely used to characterize these behaviors, providing insight into the degradation kinetics and activation energy of different fiber types. Mineral fibers such as basalt have also been extensively studied using thermal diffusivity measurements, including infrared thermography, particularly in basalt fiber–reinforced polymer composites.^145^

Basalt fibers further enhance thermal insulation performance when incorporated into composite and construction materials. Studies have shown that basalt fiber additions improve thermal insulation in foamy kaolinite-based composites, autoclaved aerated concrete, and conventional concrete mixtures, contributing to improved energy efficiency in buildings. For instance, basalt fiber–reinforced concrete has demonstrated thermal conductivity values as low as 0.283 W m^−1^ K^−1^ at a fiber content of 15%, highlighting its potential for energy-efficient structural applications.^146^

### 3.5 Morphological properties

The morphological characteristics of natural fibers play a vital role in determining their physical, mechanical, and interfacial performance in textile and composite applications. These fibers possess a complex hierarchical structure comprising multiple layers such as the primary wall, secondary wall, and lumen, which together define their surface topology, crystallinity, and overall mechanical behavior.^147^ The outer primary wall, rich in pectin and waxes, contributes to surface smoothness and protection, while the secondary wall, composed mainly of cellulose microfibrils embedded in a hemicellulose–lignin matrix, governs the fiber's strength and stiffness.^148^ The microfibrillar angle (MFA), representing the orientation of cellulose microfibrils relative to the fiber axis, is a crucial structural parameter; smaller angles (5–10°),^149^ as observed in flax and hemp, lead to higher tensile strength and stiffness, whereas larger angles in jute or coir result in improved flexibility but lower modulus.^150^ The lumen, a central hollow channel running along the fiber axis, varies in size among fiber types and influences density, moisture absorption, and thermal insulation behavior.^111^

Microscopic observations using Scanning Electron Microscopy (SEM) reveal that plant fibers generally exhibit rough and uneven surfaces with varying porosity and fibrillation, which significantly influence fiber–matrix adhesion and dye uptake.^151^ Cotton fibers show a convoluted ribbon-like structure with natural twists, flax and hemp have polygonal cross-sections with well-defined lumens,^152^ while coir fibers exhibit thick cell walls and narrow lumens contributing to higher rigidity.^152^ Protein-based fibers display distinct morphologies; wool has overlapping cuticle scales responsible for felting and frictional behavior, whereas silk filaments exhibit a smooth and continuous surface giving rise to excellent luster and drape.^153^ Techniques such as X-ray Diffraction (XRD) and Fourier Transform Infrared Spectroscopy (FTIR) are widely used to analyze crystalline orientation and molecular arrangement, confirming that most plant-based fibers possess a semi-crystalline structure dominated by cellulose I polymorph. The degree of crystallinity, which generally ranges between 50–80% depending on fiber type and treatment, directly affects tensile strength, dimensional stability, moisture regain, and dyeing properties.^154^ Thus, the morphological architecture and microstructural parameters of natural fibers are fundamental to understanding their performance and optimizing their processing for textile and composite applications.

## 4 Classification of natural fibers

### 4.1 Based on origin

**Fig. 2:**
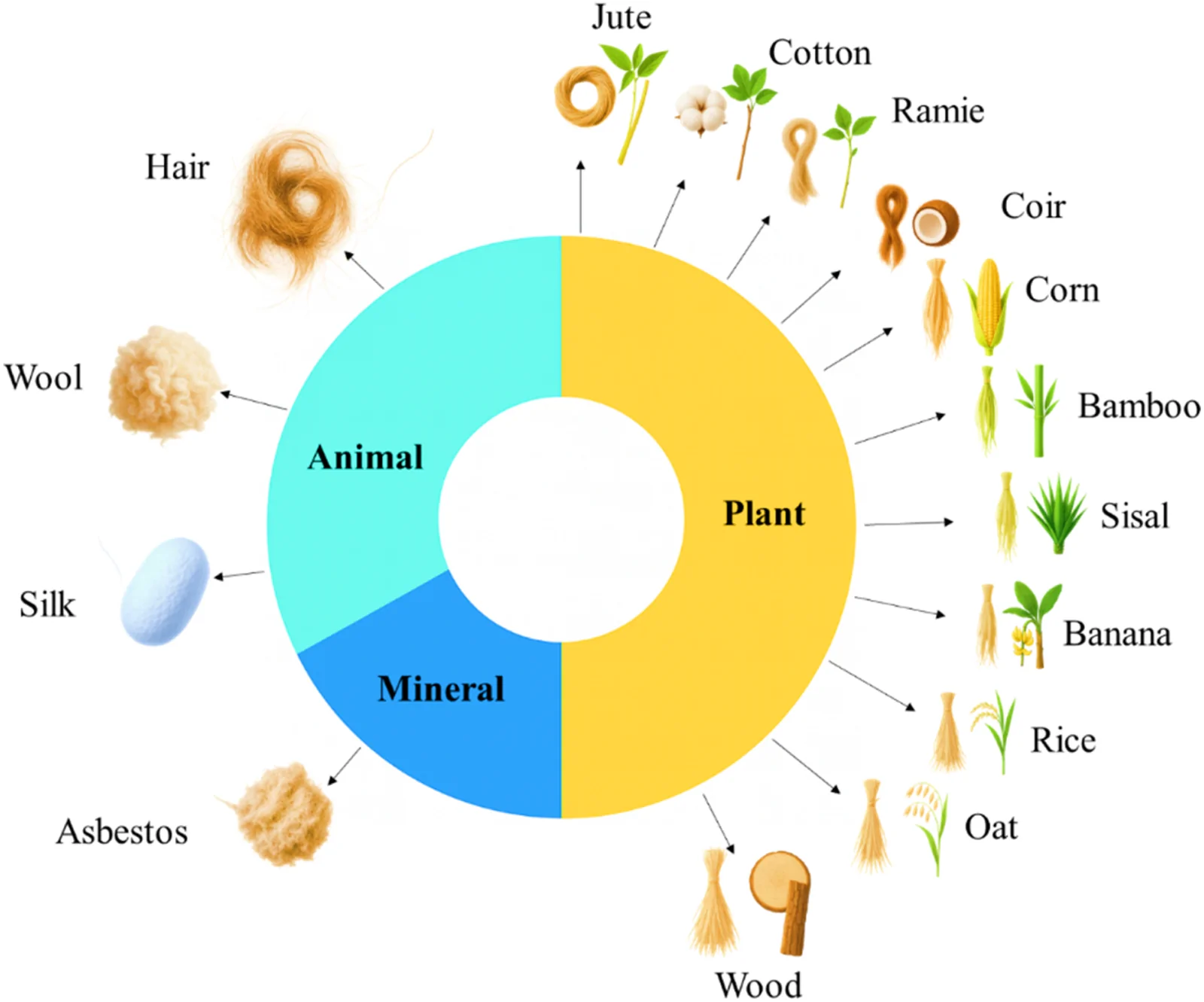
Classification of natural fibers based on origin.

This Fig. 2 illustrates the classification of natural fibers according to their source into three primary groups: plant-based, animal-based, and mineral-based fibers.

#### 4.1.1 Plant based fibers

##### 4.1.1.1 Bamboo

**Fig. 3:**
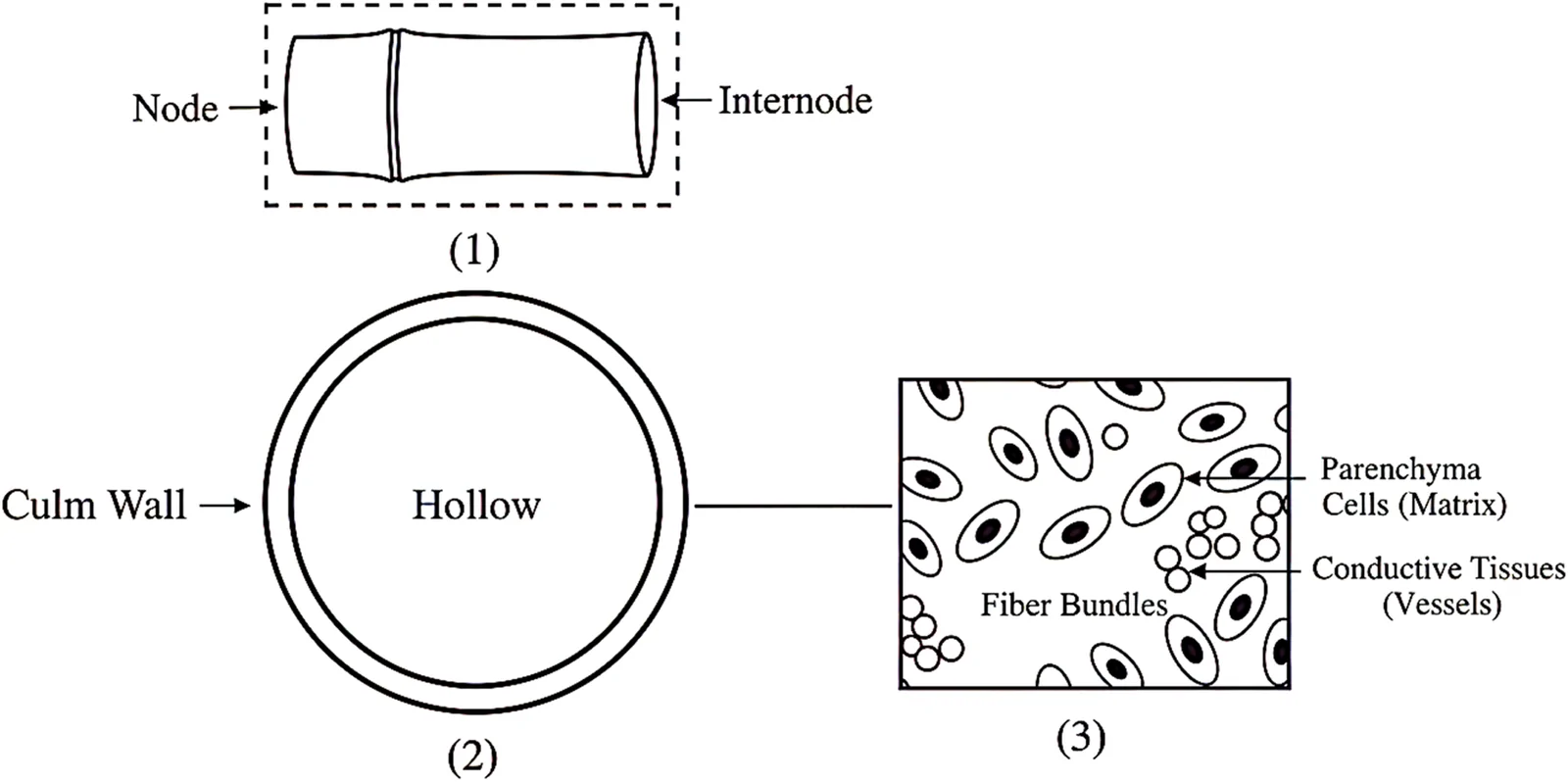
Schematic representation of the hierarchical structural organization of bamboo from the culm scale to the tissue level.

Bamboo is a fast-growing woody grass belonging to the Poaceae family and is widely distributed across tropical, subtropical, and temperate regions, particularly in Southeast Asia and South America.^155^ Its rapid regeneration, high biomass yield, and broad availability make bamboo an important renewable lignocellulosic resource for construction, furniture, textiles, and related applications. Bamboo fibers are mainly extracted from the culm, as shown in Fig. 3, and consist predominantly of cellulose, hemicellulose, and lignin, which together account for more than 90% of the dry weight, while minor constituents such as waxes, resins, tannins, proteins, and ash influence surface chemistry and processing behavior.^156^ Fiber extraction is commonly achieved through chemical or mechanical routes; chemical processing involves alkali hydrolysis with sodium hydroxide followed by carbon disulfide treatment and bleaching, whereas mechanical processing relies on enzymatic decomposition and mechanical separation. Although the mechanical route requires higher labor and cost, it is regarded as environmentally preferable due to reduced chemical consumption.^157^

Bamboo fibers have been widely examined as reinforcing agents in asphalt mixtures because of their thermal stability and fibrous morphology. Sheng *et al.*^158^ reported that bamboo fiber–reinforced dense-graded (DG) and stone matrix asphalt (SMA) mixtures maintained stability during mixing and compaction, with adequate moisture resistance confirmed by Marshall stability and freeze–thaw tests. Improvements in rutting resistance and low-temperature cracking resistance were observed, with optimal fiber contents of 0.2–0.3% for DG mixtures and 0.4% for SMA mixtures by total mixture weight. Xia *et al.*^159^ further showed that bamboo fiber can substitute lignin fiber due to superior moisture and low-temperature stability, although lignin fiber exhibited slightly higher mechanical strength. The enhanced ductility and adhesion of bamboo fiber-modified asphalt were attributed to the rough fiber surface, oil absorption capacity, and relatively high density, which contribute to improved mixture flexibility and crack resistance. Laboratory aging studies indicated that bamboo fiber-modified asphalt exhibits stiffness and fatigue behavior comparable to polyester fiber systems.^160^ However, the hydrophilic nature of bamboo can limit interfacial bonding with asphalt,^161^ prompting surface modification approaches such as melamine–formaldehyde copolymer treatment, which significantly improved tensile strength and stability, as confirmed by SEM evidence of enhanced fiber–matrix adhesion.^162^

##### 4.1.1.2 Coir

**Fig. 4:**
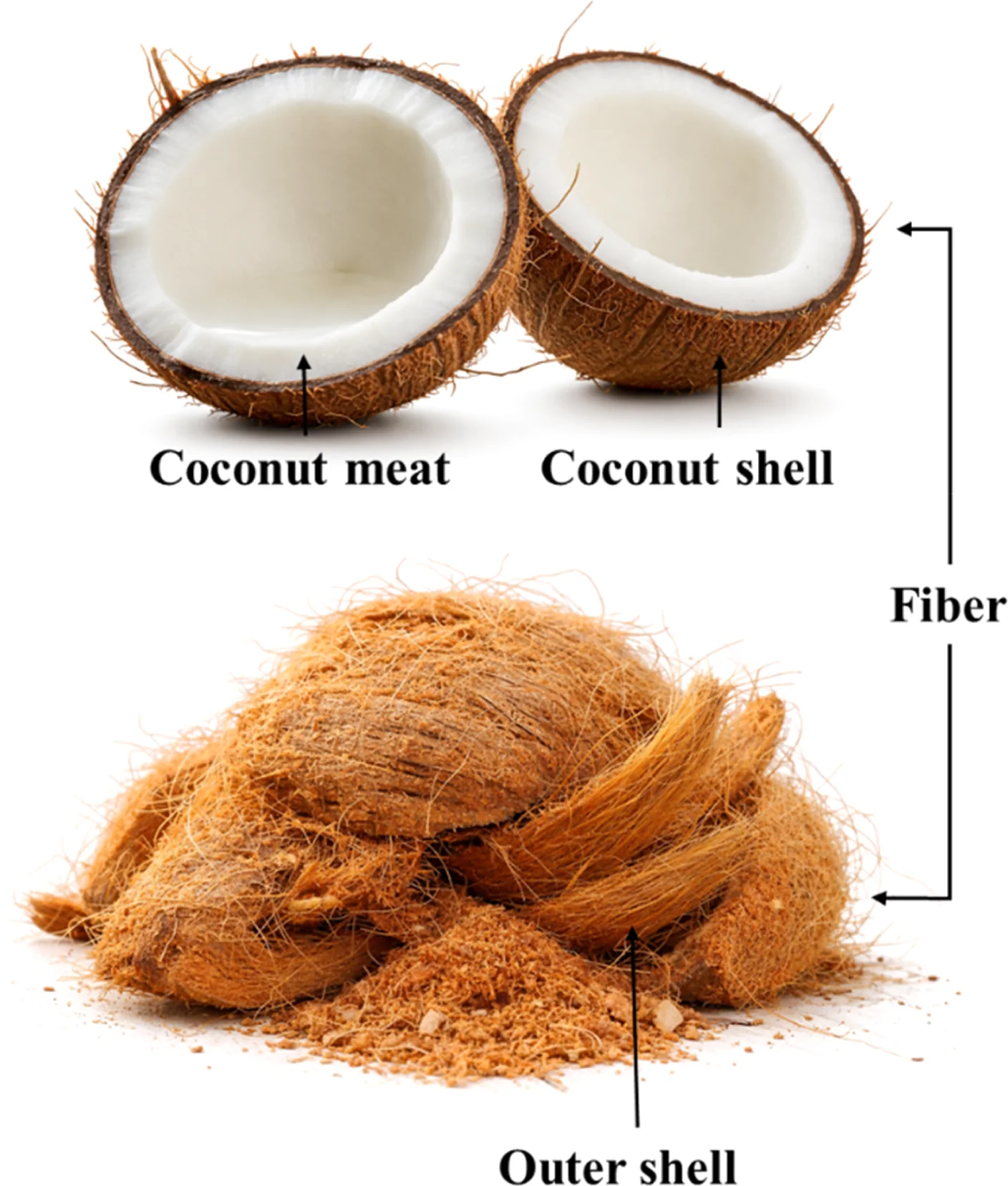
Anatomical layers of the coconut highlighting the fibrous husk used for coir fiber extraction.

Coconut fiber, also known as coir fibre, is a lignocellulosic material obtained from the husk of coconuts, which are mainly cultivated in tropical regions, particularly in Southeast Asia. Based on color, it is divided into two types: white and brown coir. The white fibres are extracted from immature coconuts, while the brown ones are obtained from fully matured fruits, as shown in Fig. 4. According to Adeniyi *et al.*,^163^ the chemical composition of coconut fibre includes cellulose (32–50%), hemicellulose (0.15–15%), lignin (30–46%), and pectin (3–4%), indicating that cellulose and lignin are its principal constituents, which provide durability, strength, and resistance to weather and water.^164^ Moreover, coconut fibre has notable advantages such as low cost, low density, high elongation at break, and biodegradability.^165^ Oda *et al.*^166^ compared coconut fibre with other natural fibres such as sisal, cellulose, and polyester and observed that coconut and sisal fibres exhibited better strength and stability characteristics. Another research also confirmed that coconut fibre could effectively replace cellulose fibre due to its superior structural properties, though its stiffness can make composites more brittle.^167^ In another investigation, Norhidayah *et al.*^168^ used both coconut shells and fibres, where the shells partially replaced coarse aggregates at varying percentages (0%, 5%, 10%, and 15%), and fibres were added at 0.3% and 0.5%. Because of their high absorption capacity, both materials were pre-treated with sodium hydroxide (NaOH) for one hour. The findings showed that combining 10% coconut shells with 0.3% coconut fibre significantly enhanced the stability and deformation resistance of the resulting material.

##### 4.1.1.3 Jute

**Fig. 5:**
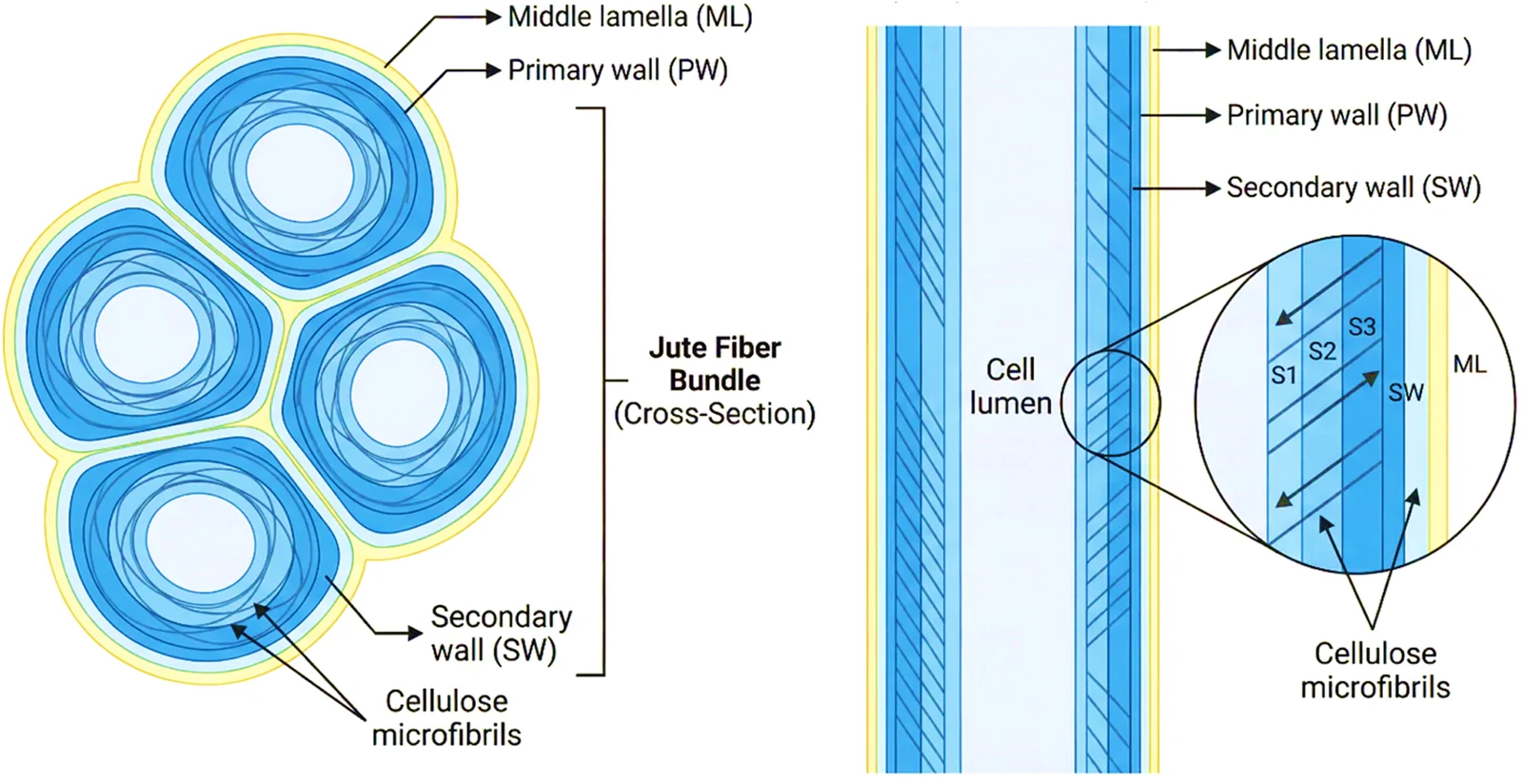
Morphological structure of jute fiber.

Jute is a bast fibre belonging to the Tiliaceae family and is primarily cultivated in countries such as India, Bangladesh, Pakistan, China, and Brazil.^169,170^ It is widely utilized in textiles, construction, and automotive industries due to its fine texture, low thermal conductivity, and affordability.^171^ Similar to other natural fibres, jute mainly consists of cellulose, hemicellulose, and lignin.^13^Fig. 5 shows the microstructure of jute fibre. Rashid *et al.*^172^ investigated the use of jute fibre as a reinforcing material, reporting that 0.5% and 1% fibre content increased stability by 29% and 10%, respectively, based on Marshall stability and flow tests, while the optimum binder content rose from 4% to 5%. The fibre-reinforced mixtures demonstrated improved deformation resistance, with 0.5% fibre identified as the most effective dosage. Ismael *et al.*^173^ examined jute, polyester, and carbon fibres at varying proportions (0.25%, 0.5%, and 0.75%) and lengths (5, 7.5, and 10 mm) through drain-down, Marshall, and wheel tracking tests. Their results showed that all fibres enhanced rutting resistance and mixture stability, particularly at 0.5% content and 7.5 mm length. Carbon fibres exhibited the highest improvement (53% in rutting resistance and 100% in dynamic stability), followed by polyester and jute fibres (34% and 63%, respectively), while jute fibres offered the best drain-down performance. In addition to their application in hot mix mixtures, jute fibres have also been successfully incorporated into warm mix^174^ and cold mix^175^ systems. For warm mix applications, the inclusion of jute fibres significantly enhanced fracture resistance under Mode I (tensile opening), indicating an improvement in tensile strength, with the optimal fibre content identified as 0.3% by total mixture weight.

##### 4.1.1.4 Sisal

**Fig. 6:**
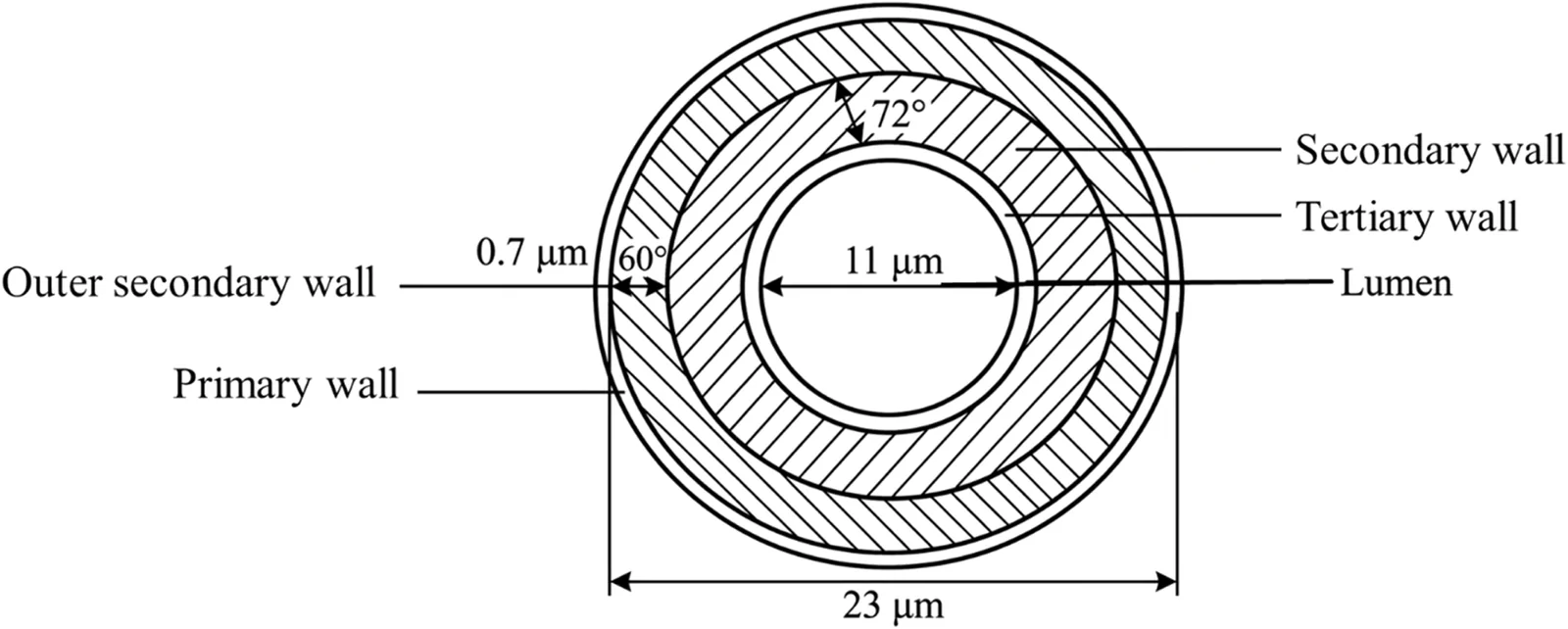
Structure of sisal fiber.

Sisal fibre is a natural leaf fibre extracted from the sisal plant, a flowering species native to southern Mexico but now cultivated widely across tropical and subtropical regions. The extraction of sisal fibre is generally carried out using two primary techniques: retting followed by scraping, or mechanical decortication.^176^ Among these, mechanical extraction tends to yield fibres of superior quality. The chemical composition of sisal fibre varies depending on the maturity of the plant; however, several studies^177^ have identified cellulose, hemicellulose, and lignin as its principal constituents, with cellulose being the most abundant. Owing to its low cost, light weight, high tensile strength, and considerable stiffness,^21^ sisal fibre has found applications in various industries, including the manufacture of paper, textiles, carpets, ropes, and geotextiles, as well as in composite and construction materials. The performance of sisal fibre-reinforced materials largely depends on the fibre length and content. Ramalingam *et al.*^178^ examined the influence of fibre lengths (5, 10, 15, and 20 mm) and contents (0.05%, 0.1%, 0.2%, and 0.3%) on the mechanical behaviour of mixtures and observed that a small addition of sisal fibre enhanced fatigue resistance and moisture durability, while excessive amounts reduced mixture stability. The best performance was achieved with 15 mm fibres at a concentration of 0.05% and an optimum binder content of 5.4%. Kar *et al.*^179^ evaluated sisal fibre as a stabilizing additive in stone mastic and dense-graded mixtures, reporting that fibre inclusion increased Marshall stability and tensile strength while reducing flow value, air voids, and drain-down. Comparatively, mixtures with 0.3% sisal fibre exhibited superior mechanical characteristics in stone mastic asphalt (SMA) than in dense-graded ones. Sisal fibre has been used in SMA mixtures containing asphalt rubber binders, where both natural fibres demonstrated better improvements in tensile strength and resilient modulus compared to polyester and cellulose fibre-reinforced counterparts.^166^Fig. 6 represented the structure of sisal fiber.^180^

##### 4.1.1.5 Cotton

**Fig. 7:**
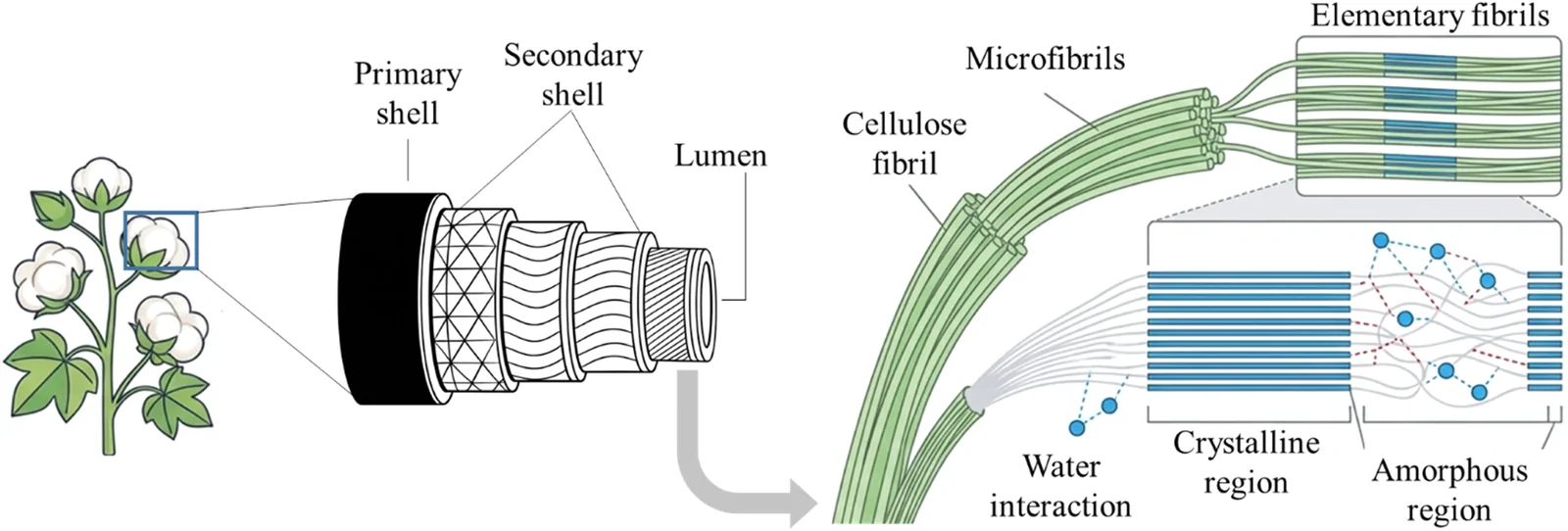
Hierarchical structure of cotton cellulose from the cell wall to the molecular level, showing cellulose fibrils organized into microfibrils and elementary fibrils with crystalline and amorphous regions.

Cotton fiber is a single-celled extension of the seed epidermis of the cotton plant (*Gossypium* genus), and it stands as the most economically significant natural textile fiber globally. It is primarily composed of cellulose, constituting approximately 88–96% of its dry weight, with the remainder consisting of proteins, waxes, and pectin.^181^ The unique properties of cotton fiber, such as its softness, absorbency, spinnability, dyeability, and comfort, contribute to its widespread use in textiles, hygiene products, and medical applications.^182^ The development of cotton fibers involves staged differentiation, including primary cell wall synthesis during elongation and subsequent secondary wall thickening, during which nearly pure cellulose is synthesized.^181^ The cellulose synthase complex (CSC), particularly the GhCesA 4, 7, and 8 subunits, plays a pivotal role in synthesizing and assembling cellulose into cell wall microfibrils, forming a 36-*mer*-like supercomplex during secondary cell wall synthesis.^183^ These microfibrils, along with elementary fibrils, constitute the structural components of the cell wall, influencing the fiber's strength and rigidity, as shown in Fig. 7.

##### 4.1.1.6 Ramie

**Fig. 8:**
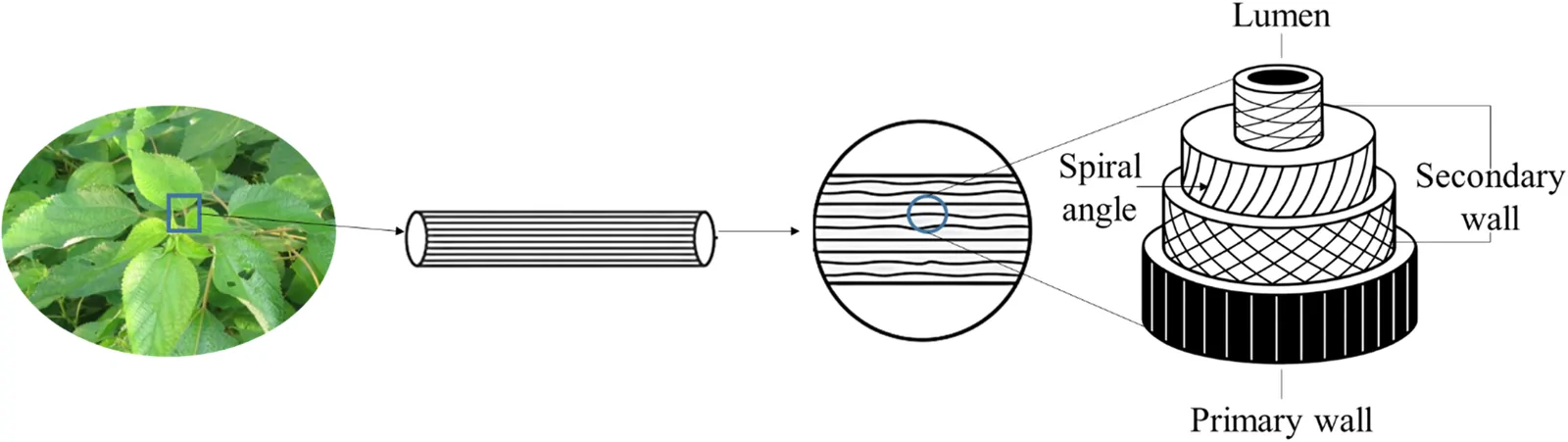
Structural representation of ramie fiber.

Ramie (*Boehmeria nivea* L.) is a perennial herbaceous plant valued for its strong bast fibers, often referred to as the “king of natural fibers”.^184^ This natural fiber possesses several desirable characteristics including attractive luster, high tenacity, enhanced strength, good microbial resistivity, and excellent moisture absorption, breathability, and antibacterial properties.^185^ These attributes make ramie a significant raw material for the textile industry and a promising candidate for various composite applications, offering a sustainable alternative to synthetic fibers.^186^ However, raw ramie fibers are tightly bound by gummy substances, which primarily consist of pectin, hemicellulose, and lignin. These non-cellulosic components must be removed through a process called degumming to separate the cellulose fibers and unveil their unique properties for textile and industrial applications.^184,187^ The efficiency of gum removal directly impacts the quality and end-use potential of ramie fibers.^188^Fig. 8 illustrated the hierarchical organization of ramie fiber.

##### 4.1.1.7 Kenaf

Kenaf (*Hibiscus cannabinus* L.) is a fast-growing annual bast fiber plant belonging to the Malvaceae family, cultivated mainly in tropical and subtropical regions such as India, Bangladesh, Thailand, and parts of Africa.^189^ It has emerged as one of the most promising renewable fibers due to its high yield potential (up to 6–10 t ha^−1^) and short cultivation period of 4–5 months.^190^ The composition of kenaf gives the fiber a combination of low density (1.2–1.4 g cm^−3^), high specific strength, and moderate stiffness, making it suitable for both textile and composite applications.^191^ The cellulose microfibrils in kenaf are oriented at a low microfibrillar angle (around 7–10°),^191^ which contributes to its high tensile strength, typically ranging from 400–800 MPa, and Young's modulus of 20–60 GPa, depending on extraction method and maturity stage.^192^

Morphologically, kenaf fibers are multicellular and polygonal in cross-section, with a rough surface texture that promotes interfacial adhesion in polymer composites. The lumen structure enhances moisture sorption and improves comfort properties when used in apparel or home textiles. However, the high hydrophilicity and presence of hemicellulose can lead to poor dimensional stability and reduced fiber–matrix compatibility, often mitigated by surface modification techniques such as alkali treatment, silane coupling, or acetylation.^131^ In addition to conventional textile uses such as ropes, canvas, and coarse fabrics, kenaf is now widely used in automotive interior panels, packaging materials, paper pulp, and geotextiles, reflecting its versatility and environmental appeal.^193^ Thermogravimetric studies indicate decomposition onset near 230–250 °C, which is adequate for most textile finishing and thermoplastic composite processing.^194^ With its favorable strength-to-weight ratio, biodegradability, and CO_2_ sequestration potential, kenaf has gained attention as a sustainable alternative to synthetic fibers and a significant contributor to the development of eco-friendly textile and industrial materials.

##### 4.1.1.8 Kapok

Kapok fiber, obtained from the fruits of *Ceiba pentandra*, is a plant-based seed fiber widely recognized for its distinctive hollow morphology and extremely low density, and is primarily cultivated in tropical regions, with Indonesia contributing approximately 85% of global supply.^195^ The fibers are unicellular, non-lignified trichomes characterized by a cylindrical lumen occupying 80–90% of the cross-sectional area, with typical lengths of 10–35 mm and diameters of 10–25 µm, resulting in a very low density of 0.03–0.05 g cm^−3^.^196–198^ The smooth fiber surface is coated with a thin wax layer, in contrast to bast fibers that show nodes or convolutions, which imparts inherent hydrophobic and oleophilic behavior.^199^ FIB-SEM analysis confirms tubular wall thicknesses of 806.1–863.3 nm and hollow ratios of 82.40–85.56%.^146^ Chemically, kapok consists mainly of cellulose, hemicellulose, and lignin, with a wax fraction of 0.5–2.0% containing hydrophobic compounds such as bis(2-ethylhexyl) phthalate and *N*,*N*-dimethyldodecanamide; surface treatments using ethanol or sodium hydroxide remove non-cellulosic components, increase surface polarity, and improve fiber–matrix adhesion.^200,201^

The pronounced hollow structure governs kapok's functional behavior, providing exceptional buoyancy and air-trapping capability but limiting tensile strength (3–8 MPa) and modulus (20–60 MPa) compared with bast fibers such as flax (345–1500 MPa).^198,202^ This structural configuration defines kapok as a low-load-bearing fiber, where performance is dominated by insulation, absorption, and lightweight functionality rather than stiffness or strength. Surface-modified kapok fibers show improved interfacial bonding in composite systems, indicating that performance can be tailored when required, although fiber orientation and lumen preservation remain critical factors.^203^ The same hollow morphology results in low thermal conductivity (0.033–0.040 W m^−1^ K^−1^) and high acoustic damping due to immobilized air within the lumen.^198^ In addition, the hydrophobic and oleophilic surface enables oil sorption capacities of 30–50 times the fiber's own weight, confirming kapok's classification as a functional fiber suited to absorption- and insulation-dominated roles rather than structural reinforcement.^204^ Consequently, kapok represents a distinct category of natural fibers where morphology-driven functionality outweighs mechanical performance, complementing bast fibers within a classification framework based on application-relevant fiber attributes.

##### 4.1.1.9 Flax

Flax fiber, obtained from the stem of *Linum usitatissimum* L., is a plant-based bast fiber widely valued for its high mechanical performance and sustainable profile. Bast fibers, including flax, hemp, jute, kenaf, and ramie, are extracted from the outer cell layers of dicotyledonous plant stems.^205^ Flax fiber extraction typically involves retting, a microbiological process that partially degrades stem tissues, followed by mechanical separation of long cellulosic fibers; traditional dew retting remains widely practiced, although process optimization is ongoing to improve fiber quality and consistency.^206^ Chemically, flax consists mainly of cellulose and hemicellulose with relatively low lignin content, along with minor pectin (1–2%) and ash (<1%). The high cellulose and low lignin content distinguish flax from wood fibers, which generally contain 25–30% lignin, contributing to superior specific mechanical properties and greater ease of chemical functionalization.^207^ Cellulose microfibrils in flax exhibit axial stiffness on the order of 100–150 GPa, exceeding that of aluminum, which underpins its performance in technical applications and phytoremediation.^208^

The hierarchical cell-wall structure governs flax's mechanical behavior, with the thick S2 secondary layer dominating performance due to cellulose microfibrils aligned at a low microfibrillar angle of 4–10° relative to the fiber axis.^209^ This arrangement enables efficient axial load transfer, resulting in tensile modulus values of 50–80 GPa and tensile strength ranging from 500 to 1500 MPa, among the highest reported for natural fibers.^210^ Flax fibers typically exhibit diameters of 12–25 µm, crystallinity indices of 65–75%, and moderate moisture regain of about 12% at 65% relative humidity, reflecting their hydrophilic nature associated with abundant surface hydroxyl groups.^207^ While hydrophilicity can reduce interfacial adhesion with hydrophobic polymer matrices, it facilitates surface treatments such as alkali, silane, or enzymatic modification, improving fiber–matrix bonding in composites.^211^ Thermally, flax fibers degrade in two stages, with hemicellulose decomposition at 200–260 °C and cellulose pyrolysis at 260–350 °C, and degradation onset above 220 °C, allowing compatibility with thermoplastics such as polypropylene and polylactic acid.^212,213^ Applications of flax span linen textiles, ropes, and geotextiles to lightweight biocomposites for automotive and construction sectors, where flax fiber–reinforced systems have shown mechanical performance comparable to synthetic fiber composites following surface treatment. Compared with other bast fibers, flax offers a balanced combination of strength, fineness, and processability, while hemp provides higher elongation, ramie higher strength but lower flexibility and more demanding processing, and jute and kenaf generally lower tensile strength.^205,214^ As a result, flax remains a benchmark bast fiber for high-performance lignocellulosic composite development.

#### 4.1.2 Animal based fibers

##### 4.1.2.1 Feather fibers

**Fig. 9:**
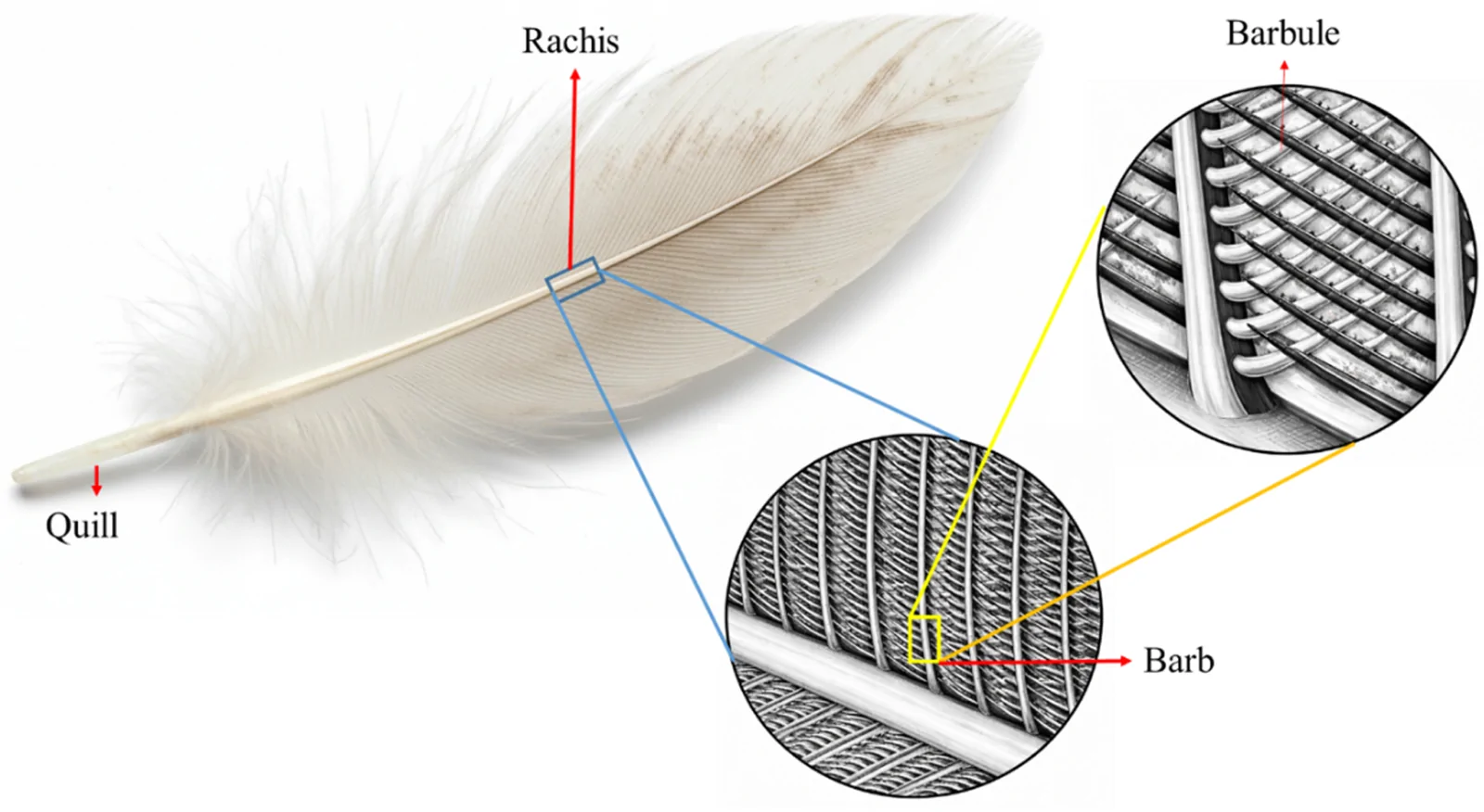
Structure of chicken feather fiber.

The poultry industry generates a substantial amount of waste, with approximately 4 billion pounds of feather waste produced annually in the United States and more than 20 billion chickens slaughtered worldwide each year.^215^ This accumulation poses environmental and health concerns, prompting growing interest in the valorization of chicken feather fibers (CFFs) as sustainable materials for composite applications, as illustrated in Fig. 9. CFFs are characterized by low density, high specific strength, and favorable thermal and acoustic insulation properties, making them suitable for lightweight and energy-efficient systems.^216^ Chemically, CFFs consist of about 91% keratin protein, 1% lipids, and 8% water, with amino acids cross-linked through disulfide and hydrogen bonds that impart mechanical integrity, chemical resistance, and durability.^217^ Structurally, feathers exhibit a hierarchical architecture comprising a central rachis with branched barbs and barbules, forming a keratin-based matrix with a honeycomb-like morphology that contributes to stiffness, flexibility, and effective sound and thermal insulation.^218^

The feasibility of incorporating CFFs into polymer composites has been demonstrated in several studies. Winandy *et al.*^219^ reported that feather fibers in wood-based composites led to a moderate reduction in stiffness but significantly improved water resistance due to the hydrophobic nature of keratin. Zhan *et al.*^220^ identified considerable variability in the tensile strength and modulus of feather barbs, enabling tailored composite design. Huda *et al.*^221^ showed that quill–polypropylene composites achieved higher noise reduction coefficients than jute-based systems, attributed to hollow quill structures and trapped air pockets, and further reported that hybrid polypropylene composites containing feather, recycled pulp, and kenaf fibers exhibited positive stiffness contributions from feather fibers, albeit lower than those of pulp fibers. Amieva *et al.*^222^ found that polypropylene composites with 5–10 wt% quill fibers optimized load transfer and dynamic storage modulus, whereas higher fiber contents resulted in matrix overloading. Cheng *et al.*^223^ demonstrated that injection-molded CFF/polylactic acid composites exhibited improved tensile modulus, storage modulus, and thermal stability compared with neat PLA, indicating effective reinforcement and interfacial adhesion. Surface modification using hydrogen peroxide, sodium hydroxide, or potassium hydroxide has further improved tensile and physicochemical properties by removing surface impurities and exposing reactive keratin sites, while Barone *et al.*^224^ reported enhanced yield stress and elastic properties in CFF/low-density polyethylene composites, supported by SEM evidence of improved fiber–matrix bonding.

##### 4.1.2.2 Silk

Silk is a protein-based natural fiber whose performance is governed by the molecular organization of fibroin. *Bombyx mori* (mulberry silk) is the most widely used form and consists mainly of fibroin and sericin, where fibroin constitutes the load-bearing filament core and sericin functions as an outer adhesive layer that is removed during processing to improve softness, luster, and dye affinity.^225^ Silk fibroin is a semi-crystalline protein polymer rich in glycine, alanine, and serine, forming β-sheet crystallites that impart tensile strength and stiffness, interspersed with amorphous domains that provide flexibility and extensibility.^226^ This hierarchical arrangement underpins silk's balance between strength and deformability. Spider dragline silk from *Nephila* species represents the upper performance limit among natural fibers, with tensile strength approaching 1.5 GPa and elongation exceeding 40%, resulting from β-sheet nanocrystals embedded in an amorphous protein matrix.^227–229^ These properties remain stable up to approximately 150 °C, while at subzero temperatures extensibility increases to about 45% without loss of tensile strength. In comparison, mulberry silk exhibits lower tensile strength, around 0.6 GPa, combined with resistance to mild acids, insolubility in most organic solvents, and low water uptake due to its compact crystalline structure, although its amorphous regions remain susceptible to hydrolytic degradation under prolonged acidic conditions.^230^ Structurally, the fibroin heavy chain comprises twelve alternating crystalline and amorphous domains, enabling controlled load transfer and elastic recovery.^231^

**Fig. 10:**
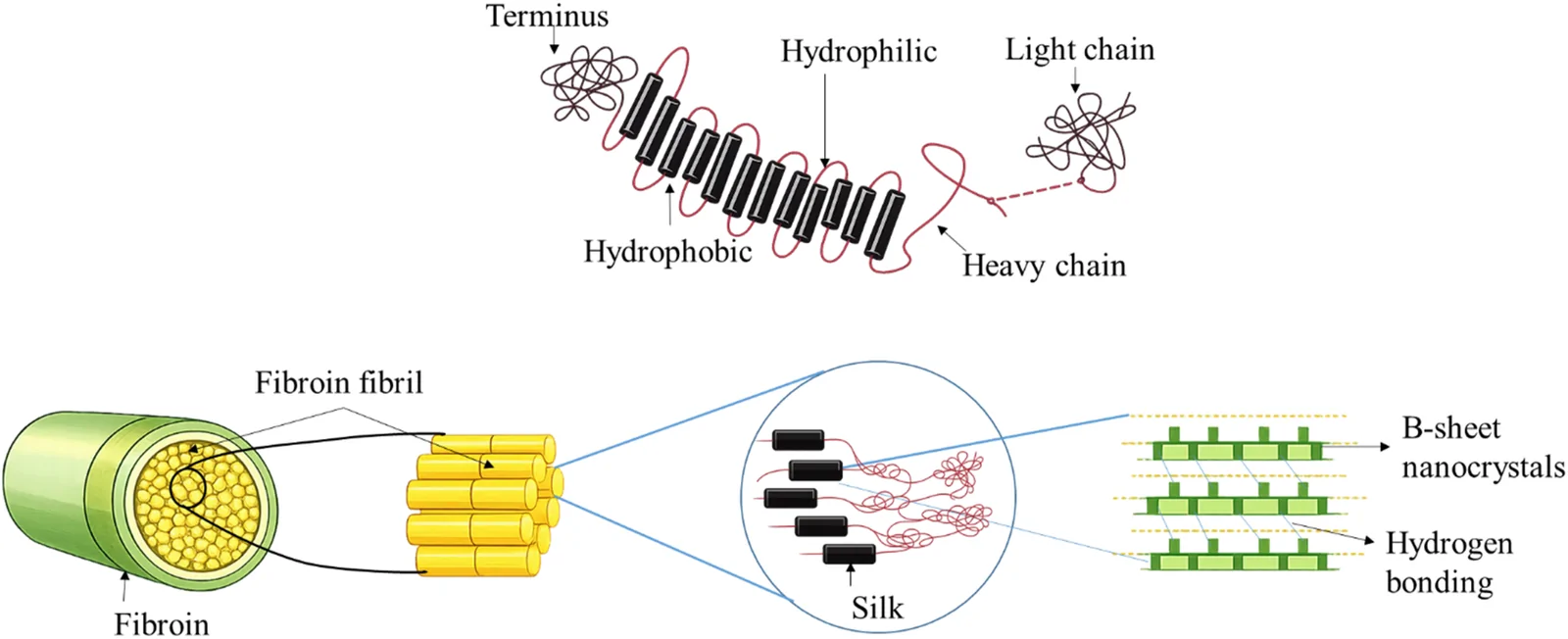
Structure of silk fiber.

Chemical and enzymatic modifications are commonly employed to adjust silk fibroin performance while preserving its protein backbone. Enzymatic grafting of acrylic acid using an H_2_O_2_–HRP system increases mechanical strength through controlled surface functionalization,^232^ while tyrosinase-catalyzed chitosan grafting improves strength and crease resistance *via* covalent bonding with oxidized tyrosyl residues on fibroin.^233^ Degradation studies consistently show preferential breakdown of amorphous regions, whereas β-sheet-rich crystalline domains remain stable, allowing predictable changes in mechanical response over time.^234^ Carbodiimide-mediated grafting further enhances fibroin reactivity and network stability through self-crosslinking and TyrP-bridged structures.^235^ These characteristics position silk as a high-toughness, protein-based natural fiber, distinct from lignocellulosic fibers, and suited to applications where flexibility, strength retention, and molecular stability are required rather than bulk structural reinforcement. Fig. 10 illustrates the hierarchical structure of silk fiber.

##### 4.1.2.3 Wool

**Fig. 11:**
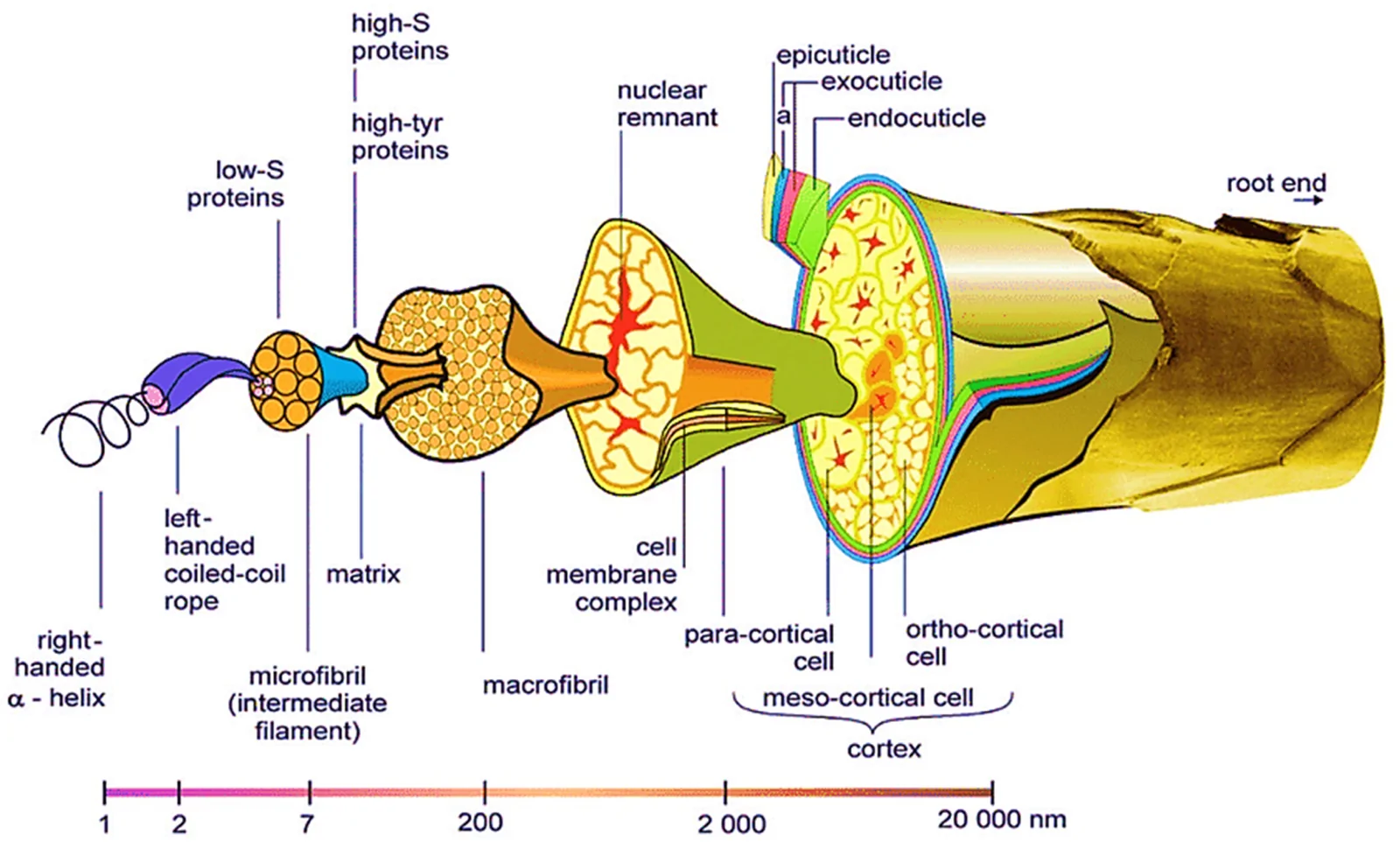
Schematic structure of wool fiber. This figure has been reproduced from ref. 244 with permission from SAGE Publications, copyright 2026.

Wool fibers are composed of keratin with a complex hierarchical structure, as illustrated in Fig. 11, where components with different properties combine to achieve functional performance.^236^ A single wool fiber is a heterogeneous biomaterial consisting of three main layers: the outer cuticle with overlapping scales, the cortex forming the primary load-bearing region, and the medulla, which may be continuous, fragmented, or absent depending on fiber type.^237^ Keratin in wool is largely insoluble in water, organic solvents, and dilute acids or alkalis due to tightly packed α-helical and β-sheet structures cross-linked by disulfide and hydrogen bonds, which impart chemical stability and resistance to degradation.^238^ Wool fiber diameter varies significantly among sheep breeds, ranging from 11.5 to 47 µm, which governs its suitability for apparel or interior textile applications.^239^ The amino acid composition, rich in cystine, serine, glutamic acid, and glycine, contributes to strength and flexibility through intermolecular bonding. Wool exhibits limited dimensional change during rewetting or swelling, typically 1–2%, due to the resistance of paracrystalline filaments. Its protein-based structure allows wool to absorb moisture up to 33% of its dry mass without feeling damp, a property attributed to hydrogen bonding within amorphous regions while crystalline domains remain largely impermeable. For fibers with approximately 25% crystallinity, the amorphous matrix alone can absorb water equivalent to about 45% of the dry weight.^240^ Sheep remain the primary source of keratin fibers for textiles, with fine wool used for clothing and coarser grades for carpets, upholstery, and insulation. Studies on wool as a building insulation material show thermal conductivity and insulation performance comparable to, or better than, mineral- and rock-based insulations.^241^ Surface modification approaches have been explored to extend wool's functionality; plasma treatment using oxygen, argon, nitrogen, or air removes the surface fatty acid layer, improving wettability and adhesion, as confirmed by SEM analysis.^242^ Enzymatic modification using transglutaminase has also been shown to enhance tensile strength and improve the stability of wool-derived keratin films in phosphate-buffered saline and simulated gastric fluid by promoting covalent cross-linking between protein chains.^243^

##### 4.1.2.4 Human hair

**Fig. 12:**
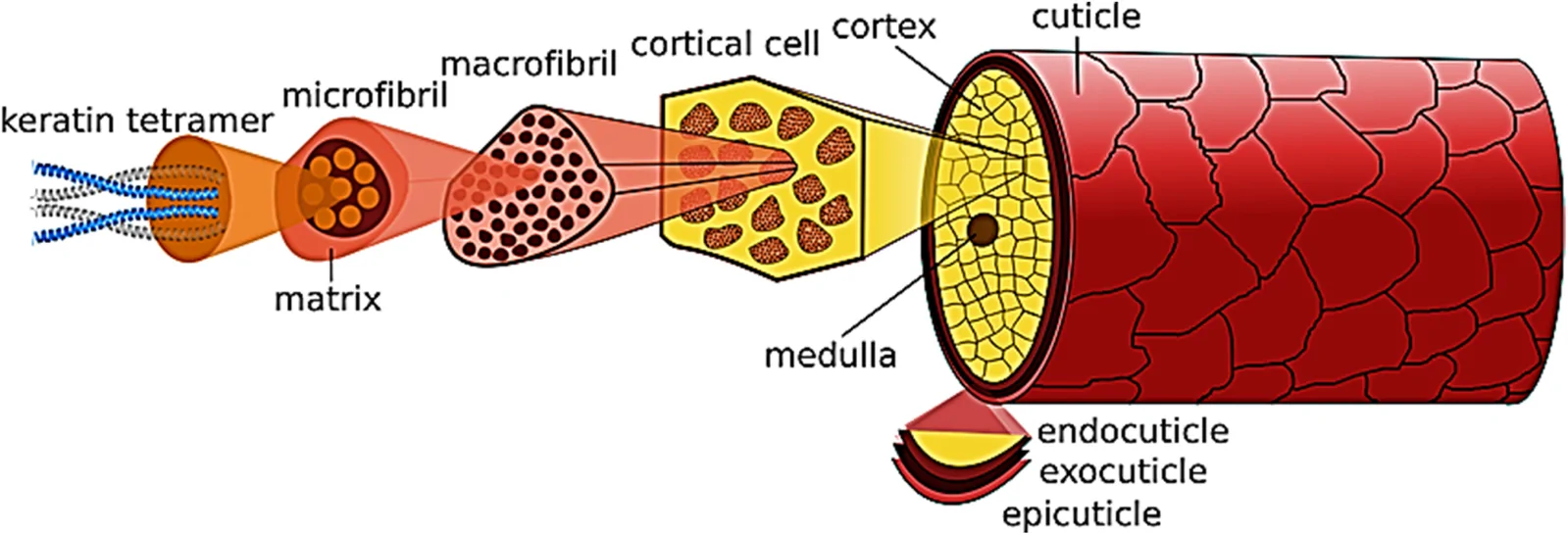
Structural components of hair fiber. This figure has been reproduced from ref. 247 with permission from MDPI, copyright 2016.

Human hair, an abundant yet often underutilized non-biodegradable waste material, has recently gained attention as a potential reinforcement fiber for bio-composite and engineering applications. Despite being discarded in large quantities worldwide, human hair exhibits favorable mechanical properties, including a surface tensile strength ranging from 150 to 220 MPa, making it a strong and flexible natural fiber. The primary component of human hair is keratin, a fibrous structural protein composed of long chains of amino acids forming high-molecular-weight polymers.^245^ These keratin proteins constitute approximately 65–95% by weight of the total fiber mass and are responsible for its mechanical strength, flexibility, and chemical resistance. Fig. 12 illustrates the key structural components of human hair fiber, including the cuticle, cortex, and medulla, which collectively contribute to its performance characteristics.^246^

**Fig. 13:**
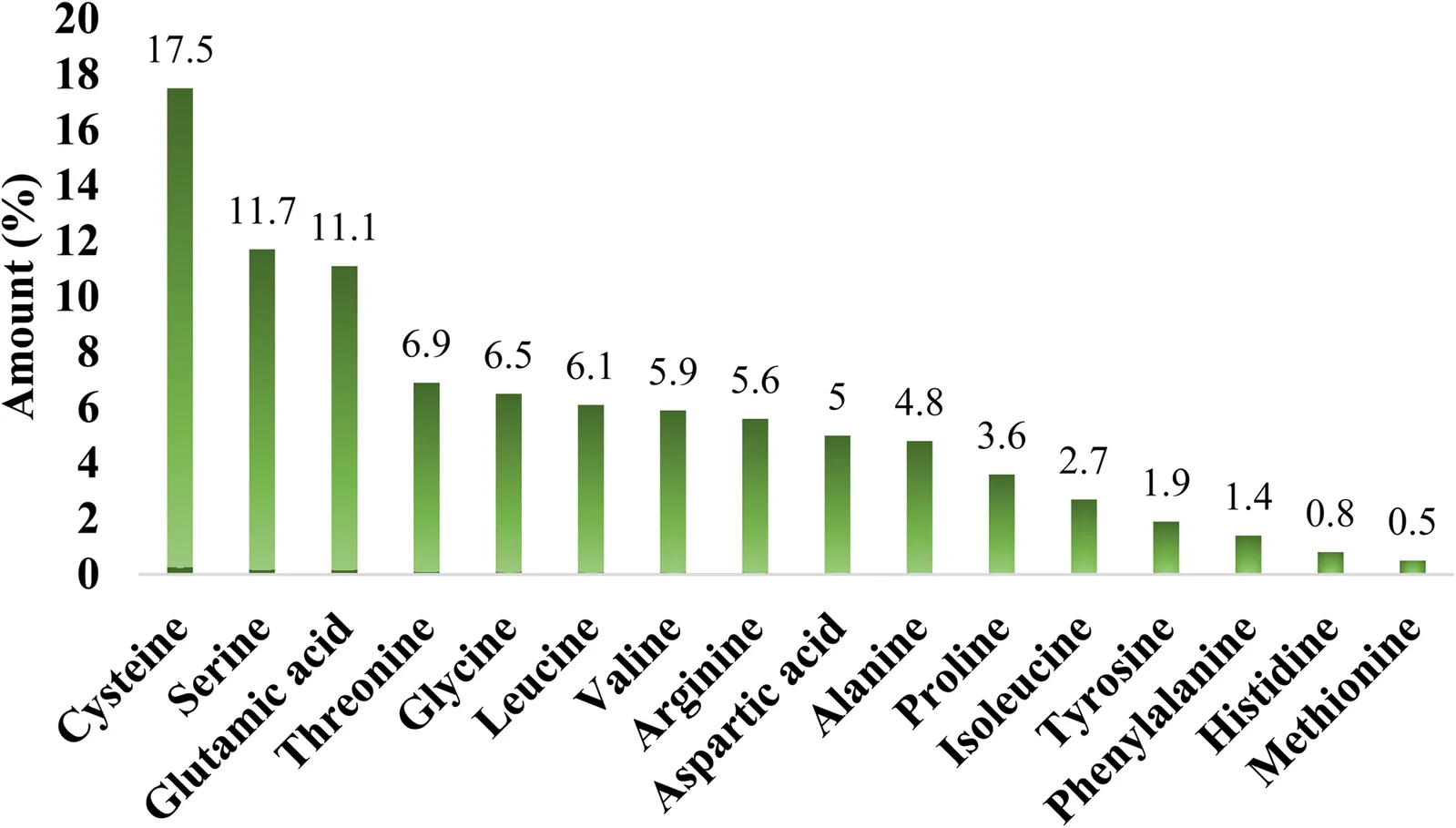
Distribution of amino acids based on their relative abundance.

The reinforcing potential of human hair in polymer composites has been demonstrated in several studies. Rao *et al.*^248^ investigated polyester composites reinforced with human hair at different fiber volume fractions and observed that lower fiber contents led to intra-fiber voids, whereas higher contents resulted in inter-fiber voids, highlighting the importance of fiber dispersion and interfacial bonding. An optimal fiber volume fraction yielded a maximum tensile strength of 23.5 MPa, confirming that processing conditions strongly influence composite performance. The combination of rigidity and flexibility associated with cortex keratin makes human hair suitable for applications requiring impact resistance and ductility.^249^ Hair properties are further influenced by intrinsic and extrinsic factors such as amino acid composition, age, environmental exposure, and chemical treatments, as thermal and mechanical stresses can alter hydrogen bonding and disulfide linkages within the α-helical keratin structure.^240^ While the cortex governs tensile response, non-keratin components of the cuticle and the cell membrane complex contribute to fiber cohesion and resistance to surface wear during grooming and mechanical loading.^250^ The amino acid profile of human hair, shown in Fig. 13, reflects the dominance of amino acids responsible for structural stability, elasticity, and moisture-binding behavior.

#### 4.1.3 Mineral based fibers

##### 4.1.3.1 Asbestos

Asbestos is a naturally occurring silicate mineral fiber historically recognized for its fibrous morphology, high tensile strength, chemical inertness, and exceptional resistance to heat and fire.^251^ It represents a classical example of mineral-based natural fibers and was extensively used in industrial applications during the 20th century. Asbestos fibers are grouped into two mineral families, namely serpentine and amphibole.^252^ The serpentine group consists mainly of chrysotile (white asbestos), characterized by curly and flexible fibers, while the amphibole group includes amosite, crocidolite, tremolite, anthophyllite, and actinolite, which exhibit straight, needle-like, and more brittle morphologies.^253,254^ Chemically, chrysotile is composed of hydrated magnesium silicate (Mg_3_Si_2_O_5_(OH)_4_), whereas amphibole asbestos contains calcium–magnesium–iron silicates.^255^ The fibrous habit originates from the crystalline arrangement of silica tetrahedra into chain-like structures that allow longitudinal splitting into fine fibrils.

Asbestos fibers exhibit very high tensile strength, typically in the range of 700–3000 MPa, thermal stability up to approximately 1000–1200 °C, and strong resistance to most acids and alkalis.^251^ Their density ranges from 2.2 to 2.7 g cm^−3^ depending on mineral type.^256^ The extremely high aspect ratio, often exceeding 100 : 1, enabled efficient stress transfer and underpinned their historical use in cement composites, insulation materials, brake linings, roofing sheets, gaskets, and friction products.^257^ Chrysotile asbestos accounted for nearly 90% of global consumption due to its flexibility, fine fibrillar structure, and strong interlocking capability with cementitious and polymer matrices, resulting in improved strength, dimensional stability, and heat resistance.

The thermal and acoustic insulation performance of asbestos arises from its low thermal conductivity, high specific surface area, and porous fibrous network, which made it effective in high-temperature environments. However, due to its well-established health risks, including severe respiratory diseases associated with fiber inhalation, asbestos mining, processing, and use have been banned or strictly regulated in many regions.^258^ Consequently, research has shifted toward safer alternative mineral fibers such as basalt, wollastonite, and sepiolite that aim to replicate asbestos-like thermal endurance and mechanical performance without associated health hazards.^259^ Despite its prohibition, asbestos remains a benchmark mineral fiber in materials science, serving as a historical reference point for evaluating and designing non-toxic inorganic reinforcements with comparable durability and heat resistance.

##### 4.1.3.2 Wollastonite

Wollastonite is a naturally occurring calcium silicate mineral (CaSiO_3_) that crystallizes in a triclinic structure and typically develops as acicular or needle-like crystals during the metamorphism of siliceous limestone.^260^ It is a non-toxic mineral-based natural fiber known for its fibrous morphology, excellent mechanical strength, and high chemical and thermal stability.^111^ The fibers of wollastonite generally have diameters ranging from 2 to 20 µm and lengths between 10 and 100 µm, with aspect ratios of 5 : 1 to 20 : 1, depending on the refining process.^261,262^ Wollastonite exhibits a density of about 2.9 g cm^−3^, tensile strength of 20.45 MPa, and a bending strength is 38.02 MPa, placing it within the performance range of conventional inorganic fibers used in technical textiles.^263^

The acicular morphology of wollastonite fibers contributes significantly to their reinforcing behavior in textile composites and high-performance fabric structures.^264^ When incorporated into textile-reinforced polymer matrices or nonwoven technical textiles, wollastonite enhances dimensional stability, rigidity, and thermal resistance while maintaining low density and high whiteness.^265^ Its high aspect ratio enables efficient stress transfer within the fiber matrix, improving fabric stiffness, abrasion resistance, and durability under thermal and mechanical loading. The fibrous particles can also act as functional fillers in coated and laminated textiles, where they improve flame retardancy, UV resistance, and thermal shielding performance without compromising flexibility or texture.^262^ In protective and industrial textiles, wollastonite fibers are used in combination with glass or aramid fibers to enhance the heat barrier properties of woven and nonwoven composites.^266^ Their high refractoriness and resistance to molten metal splashes also make them suitable for heat-protective suits, welding aprons, and furnace insulation cloths.

In addition to performance textiles, wollastonite fibers are being explored in eco-friendly fabric coatings and specialty nonwovens as mineral fillers or reinforcement materials that reduce flammability and improve dimensional integrity. Their natural whiteness and smooth surface allow uniform dispersion in polymeric textile coatings such as polyurethane (PU) and silicone matrices, enhancing heat stability and wear resistance of coated fabrics.^267^ Moreover, surface modification treatments, including silane coupling or acid etching, can be employed to improve the adhesion of wollastonite with organic binders and textile resins, leading to composites with enhanced mechanical and thermal behavior. These features make wollastonite an increasingly important functional mineral fiber in the development of advanced, sustainable technical textiles and heat-resistant fabric systems.^268^

##### 4.1.3.3 Basalt

Basalt fiber (BF) is a high-performance inorganic fiber produced by melting and continuously extruding natural basalt rock, typically sourced from volcanic formations, at temperatures of approximately 1450–1500 °C.^269^ Owing to its geological origin and silicate-based composition, BF is classified as a mineral fiber, although it is frequently discussed alongside natural fibers in composite and sustainability studies.^269^ Unlike plant- or animal-derived fibers, BF is intrinsically inorganic and consists primarily of SiO_2_, Al_2_O_3_, CaO, MgO, and iron oxides, with minor amounts of Na_2_O, K_2_O, and TiO_2_, which contributes to its compatibility with cementitious systems.^270^ At the atomic level, BF is formed from interconnected silicon–oxygen tetrahedra that generate a chemically stable and non-fibrous structure, in contrast to the chain-like crystalline arrangements of amphibole asbestos responsible for its hazardous morphology.^271^ Basalt fibers typically exhibit diameters in the range of 10–25 µm.^269^

Mechanically, BF exhibits very high tensile strength (approximately 3000–4840 MPa) and elastic modulus (70–95 GPa), exceeding those of many lignocellulosic fibers such as hemp and sisal and approaching the performance of E-glass fibers.^270^ These properties enable BF to significantly enhance compressive, flexural, and tensile behavior in cement-based and polymer composites, with optimal fiber contents improving strength, crack resistance, and durability.^272^ Hybrid systems combining basalt fibers with natural fibers such as jute have further demonstrated synergistic reinforcement effects.^270^ Thermally, BF is non-combustible (LOI > 80%), remains dimensionally stable up to about 700 °C, and decomposes only above 1000 °C, making it far superior to organic fibers that degrade or ignite between 200 and 300 °C.^269,270^ BF also shows good resistance to weak acids, salts, and weathering; however, limited alkali resistance in highly alkaline environments such as concrete has prompted the development of alkali-resistant basalt fibers and compositional modifications.^273^ These combined properties have led to widespread use of BF in construction (HPC, UHPC, ECC, recycled concrete), automotive and aerospace composites, fire-resistant insulation, gypsum and asphalt systems, and lightweight structural components.^274^ Importantly, BF is now recognized as a safer, environmentally friendly alternative to asbestos in high-temperature and insulation applications, offering comparable thermal and mechanical performance without the severe health risks associated with asbestos exposure.^270^

### 4.2 Based on application

Natural fibers can be classified according to their end-use applications, which reflect their intrinsic physical, mechanical, chemical, and aesthetic properties. The performance of a fiber in a particular textile product depends on parameters such as tensile strength, fineness, flexibility, moisture absorption, dye affinity, and comfort-related characteristics, as demonstrated in Table 5. Each category employs different fiber types and processing techniques to meet specific functional or aesthetic requirements.

**Table 5:** Application-based classification of natural fibers according to dominant functional attributes

Application class	Defining structural/chemical attributes	Representative fiber types	Functional mechanisms	Typical performance indicators
Structural load-bearing fibers	High cellulose crystallinity, low microfibril angle, aligned cellulose microfibrils, high axial stiffness	Flax, hemp, jute, kenaf, ramie	Efficient axial stress transfer through parallel cellulose microfibrils	High tensile strength, high modulus, low elongation
Thermal and acoustic insulation fibers	High hollowness, high porosity, low density, air-trapping morphology	Kapok, coir, luffa, wood fibers, wool, feather fibers	Suppression of heat conduction and convection, acoustic damping *via* trapped air	Low thermal conductivity, high sound absorption
Comfort and apparel fibers	Fine fiber diameter, low bending rigidity, moisture sorption and transport capability	Silk, cotton, merino wool, bamboo fibers	Soft handle, drape control, hygroscopic buffering, vapor transport	High softness, moisture regulation, skin comfort
Protective and high-temperature-resistant fibers	Thermal stability, flame resistance, chemical inertness, char-forming capability	Basalt fiber, wool, flame-retardant cotton, treated ramie	Resistance to heat and flame, char formation, flame-retardant surface chemistry	High limiting oxygen index, thermal stability, fire resistance
Sorption, filtration, and separation fibers	Controlled lumen size, surface chemistry, pore hierarchy	Kapok, modified cellulose fibers, agricultural residues	Capillary entrapment, selective permeation, affinity-based separation	High oil uptake, selective separation, filtration efficiency

#### 4.2.1 Load-bearing and structural natural fibers

Structural and load-bearing natural fibers are primarily represented by plant bast fibers such as flax, hemp, jute, kenaf, and ramie, which are distinguished from other natural fibers by their high specific stiffness and strength.^205^ These fibers share a common cell-wall architecture characterized by high cellulose crystallinity (often >70%) and a low microfibril angle, typically below 10°, resulting in cellulose microfibrils aligned nearly parallel to the fiber axis.^205,275^ Compared with other plant fibers, this parallel alignment within a lignin–hemicellulose matrix enables more efficient axial stress transfer and underpins their classification as structural fibers.^205^

Within this group, flax is often used as a reference bast fiber due to its high tensile strength and stiffness, which arise from the highly crystalline and well-aligned cellulose microfibrils in the thick secondary walls of sclerenchyma cells.^276^ Hemp exhibits comparable load-bearing capability, combining high tensile strength with a relatively high Young's modulus, while kenaf and jute provide structurally useful reinforcement with greater sensitivity to processing conditions and moisture uptake, respectively.^277^ Across all bast fibers, lignin in the secondary wall acts as a natural binding phase, filling spaces between cellulose and hemicellulose to form a rigid, compression-resistant structure analogous to reinforced concrete, contributing to dimensional stability under moderate hygrothermal exposure.^205^

In contrast, most animal-derived fibers are excluded from this structural classification. Although silk fibers exhibit high toughness and β-sheet-driven crystallinity, limitations related to thermal stability above 150 °C, scalability, and interfacial compatibility restrict their use to specialized or hybrid reinforcement roles rather than primary load-bearing functions.^278^ Keratin-based fibers such as wool are similarly excluded due to low stiffness and high hygroscopicity. It is notable, however, that animal biological systems rely on collagen fibers within extracellular matrices to resist tensile loads, serving a structural role analogous to cellulose microfibrils in plant bast fibers.

Mineral fibers, particularly basalt fiber, form a separate class of load-bearing natural fibers. Unlike biologically derived fibers, basalt fiber is produced from molten basalt rock and exhibits homogeneous filament morphology, negligible moisture absorption, and high axial stiffness derived from strong Si–O and metal–oxygen bonding networks.^279^ When compared with bast fibers, basalt fiber offers superior thermal and chemical stability, long-term dimensional predictability, and creep resistance, positioning it alongside plant bast fibers in structural classifications while distinguishing it by durability and environmental robustness.^280^

#### 4.2.2 Thermal and acoustic insulation natural fibers

Natural fibers classified for thermal and acoustic insulation are defined by structural features such as hollowness, high porosity, and low bulk density, which collectively promote air entrapment and limit heat transfer and sound propagation. Hollowness, referring to internal lumens within fibers, reduces solid-phase thermal conduction by replacing dense material with air, a poor heat conductor.^142^ Kapok fiber (*Ceiba pentandra*) represents the most pronounced example, exhibiting a tubular morphology with thin cell walls of approximately 806.1 nm and hollow ratios exceeding 85%, resulting in extreme lightness and effective thermal insulation.^196^ Similar insulation behavior is observed in luffa fibers, whose sponge-like hollow structure enhances both thermal retention and acoustic damping,^281^ and in bio-inspired fibrous systems modeled after polar bear hair, where internal air pockets dominate insulation performance.^282^

Porosity further governs this classification by describing void space generated within individual fibers and across fibrous assemblies. Highly porous networks trap air in interconnected pathways, suppressing convective heat transfer and dissipating sound energy through viscous and thermal losses. In kapok-based fibrous materials, sound absorption improves as porosity is optimized at low bulk density,^283^ while highly porous ligno-nanocellulosic foams demonstrate that ultralow density is closely linked to effective insulation.^284^ Low density therefore arises directly from the combined effects of hollowness and porosity, with extensive air voids reducing thermal conductivity by limiting the solid fraction available for heat conduction.^197^ This air-trapping mechanism is further reinforced by tortuous fiber networks that restrict airflow and inhibit convective heat transport.

Within plant fibers, kapok is the most insulation-oriented due to its high hollowness, fineness, wax-coated surface, and hydrophobicity, enabling its use in nonwoven fabrics, composite aerogels, and blended systems with polymers or biopolymers such as polypropylene and chitosan.^197^ Coir fibers, although less hollow, are highly lignified and form rigid open-pore networks that contribute to thermo-acoustic performance and have been combined with kapok in sustainable concrete systems to improve insulation and pore structure.^285^ Luffa fibers offer an intrinsically porous structure suitable for insulation panels,^281^ while agricultural residues such as corn husk fibers and wood fibers exploit cellular morphologies for sound absorption and thermal regulation.^286,287^ Hemp and other cellulose fibers, typically classified for reinforcement, also fall within this insulation category when used in porous assemblies due to their internal pore structure and renewable origin. Marine fibers from Posidonia oceanica further extend this group, with loose-fill bulk densities ranging from 17 to 155 kg m^−3^, indicating suitability for lightweight thermal insulation.^288^

Animal fibers form a parallel insulation class based on hierarchical microstructures rather than large lumens. Wool fibers generate stable air pockets through natural crimp and surface complexity, supporting thermal and acoustic insulation in standalone materials and in hybrid systems blended with agricultural residues such as sugarcane bagasse.^281^ Feather fibers derived from poultry waste exhibit a branched architecture with hollow quills and barbules, producing lightweight, highly porous assemblies that effectively trap air while offering a sustainable route for waste valorization.^289^ Although not biologically derived, mineral fibers such as rock wool and porous mineral aggregates including pumice stone powder display analogous insulation behavior through fibrous skeletons and air-filled pore networks, justifying their inclusion as structural analogues within this insulation-based classification.^290^

#### 4.2.3 Comfort-oriented and apparel-grade natural fibers

Natural fibers classified for comfort and apparel applications are distinguished by fineness, flexibility, moisture management, surface smoothness, and skin compatibility, all of which govern drape, handle, and thermo-physiological comfort.^76^ Fiber fineness, commonly expressed as diameter in micrometers, plays a central role, as finer fibers generally yield softer and more flexible textiles. Silk exhibits the finest natural filament diameter, typically 10 to 13 µm, which accounts for its characteristic drape and luxurious hand feel. Merino wool, valued for next-to-skin comfort, usually has mean fiber diameters below 21.5 µm, whereas coarser wool fibers exceeding 25 µm tend to induce prickle.^76,291^ Cotton occupies an intermediate range, typically 12 to 22 µm, providing a balance between softness and durability, while flax fibers are comparatively coarser at 12 to 50 µm and may feel stiff unless appropriately processed.^292^

Flexibility, closely related to bending rigidity and influenced by both fiber diameter and modulus, further differentiates apparel-grade fibers. Silk exhibits low bending resistance due to its β-pleated sheet molecular structure, resulting in smooth drape, while wool derives flexibility and resilience from its natural crimp and surface scales.^76^ Cotton's twisted ribbon morphology contributes to its pliability, whereas flax, due to higher crystallinity and lignin content, is inherently stiffer but can be rendered suitable for apparel through treatments such as enzymatic retting and mercerization.^293^ Moisture management is another defining criterion, encompassing hygroscopicity, wicking, and evaporative efficiency. Wool demonstrates exceptional dynamic moisture regulation by absorbing up to 30% of its weight in moisture vapor without feeling wet, maintaining thermal comfort even in damp conditions. Cotton shows high hygroscopicity with around 8% moisture regain and effective capillary wicking, while bamboo fibers, often used in blends, enhance spreading and wetted area in fabrics.^294^ Silk, though less effective in liquid wicking, allows efficient moisture vapor transmission. The hydrophilic nature of many plant fibers supports comfort in apparel but can require chemical modification when interfacing with hydrophobic polymer systems.^295^

Softness and skin compatibility further refine this classification. Silk exhibits very low surface friction, with coefficients around 0.15, producing a smooth and cool touch, while ultrafine merino wool minimizes prickle through reduced fiber diameter and bending stiffness.^296^ Cotton is widely regarded as skin-friendly due to its smooth cuticle and low irritation potential, whereas regenerated cellulose fibers such as lyocell and viscose offer enhanced softness through uniform fiber morphology.^297^ This comfort-oriented classification differs from botanical or structural groupings of fibers. Although fibers may be broadly categorized as plant-based, animal-based, or mineral-based, such classifications do not directly reflect tactile or physiological comfort. For example, cotton and lyocell are both cellulose-based but differ markedly in fibril orientation and surface morphology, leading to distinct comfort behavior, while lignocellulosic fibers such as bamboo, kenaf, and sugar palm exhibit varied microstructures that influence their textile performance.^296^ Consequently, this category groups fibers according to comfort-related functional attributes rather than origin or load-bearing capability.

#### 4.2.4 Functional and protective natural fibers

Protective and high-temperature-resistant natural fibers are characterized by their ability to maintain functional performance under thermal loading, flame exposure, and chemically aggressive environments. Among these materials, mineral fibers, particularly basalt fiber, exhibit the highest level of inherent thermal and chemical stability due to their inorganic silicate composition.^269,298^ Basalt fibers show low thermal conductivity, remain thermally stable up to approximately 800 °C, and possess melting temperatures exceeding 1000 °C, while being non-flammable and resistant to acids, alkalis, and ultraviolet radiation.^269,298^ These characteristics explain their extensive use in cementitious composites, structural elements subjected to thermal cycling, and braking systems where frictional stability and durability at elevated temperatures are required.^299^ In addition to intrinsic properties, surface modification techniques such as magnetron sputtering with aluminum or zirconium dioxide coatings have been reported to further improve resistance to radiant and contact heat, while preserving the fundamental thermal and chemical stability of the basalt fiber itself.^300^

In contrast to mineral fibers, animal-derived fibers achieve thermal protection through different physicochemical mechanisms. Keratin-based fibers such as wool exhibit inherent flame resistance associated with their nitrogen- and sulfur-rich protein structure, which promotes the formation of a stable char layer during thermal exposure and suppresses flame propagation.^46^ This behavior supports their use in protective textile systems where fire safety and moderate thermal resistance are required. However, the temperature tolerance and long-term thermal durability of keratin fibers remain lower than those of basalt fibers, which limits their suitability to applications involving less severe thermal conditions.

Plant-derived fibers further extend this progression, as their intrinsic thermal stability is generally lower, but their performance can be significantly modified through chemical treatments. Cotton, for example, exhibits a limiting oxygen index of approximately 18 percent and ignites readily in its untreated state.^301^ To address this limitation, flame-retardant behavior has been introduced through chemical grafting, chelation, and coatings based on phosphorus-containing compounds, lignin, and hybrid nanoparticle systems, which impart durable flame retardancy along with auxiliary functions such as ultraviolet shielding and water repellence.^212^ Comparable strategies have been applied to other lignocellulosic fibers such as ramie and lyocell using phosphorus-based systems and bio-derived layer-by-layer assemblies to enhance flame-retardant and antibacterial properties.^302^ Together with surface treatments such as mercerization and alternative approaches including microwave processing, these modifications improve thermal stability, durability, and compatibility with polymer matrices, enabling plant fibers to satisfy fire-safety requirements in composite systems alongside mineral and animal fibers.^303^

#### 4.2.5 Sorption, filtration, and separation-oriented natural fibers

Natural fibers used for sorption of nonpolar liquids are distinguished by large hollow lumens, typically greater than 10 µm, hydrophobic surface chemistry, and low intrinsic wall porosity, which together enable efficient oil uptake through capillary entrapment and surface adsorption with minimal flow resistance.^304^ Kapok fiber (*Ceiba pentandra*) is a representative material in this group, as its pronounced hollow tubular structure and waxy cutin layer impart high hydrophobicity and oleophilicity, supporting effective oil spill remediation and oil–water separation.^304^ Sorption capacity depends on packing density and surface chemistry, with untreated kapok fibers reported to achieve oil sorption up to 20.72 g g^−1^.^305^ Surface modifications such as calcium stearate or ZnO coatings further enhance sorption efficiency and reusability by altering surface roughness and chemical affinity.^4,9^ Other naturally hollow fibers, including desert rose seed fibers, exhibit similar sorption behavior due to comparable morphology.^306^

Filtration-oriented fibers rely on different structural features, including small or collapsed lumens, generally below 2 µm, high surface hydrophilicity, and abundant surface-wall microporosity, which facilitate selective retention of particles or molecules.^307^ Filtration performance is governed by pore size distribution and internal surface area, often characterized by nominal or absolute pore ratings and quantified using BET analysis. Cellulose-based membranes illustrate this behavior by acting as selective barriers based on size exclusion, charge, and polarity, although membrane fouling remains a key limitation.^308^ In this context, modified kapok fibers coated with polyaniline demonstrate that surface chemistry manipulation can shift inherently sorptive fibers toward selective filtration functions, while carbon aerogels derived from kapok and regenerated cellulose provide controlled porosity suitable for oil–water filtration.^309^

Separation-oriented fibers extend these principles by combining controlled pore hierarchy with tunable surface chemistry to enable affinity-driven partitioning and selective permeation. Functionalized nanofibers bearing specific diammonium reagents have been used for selective recovery of iridium from rhodium, highlighting the role of tailored surface chemistry in separation processes.^310^ Surface chemistry also governs fouling resistance and separation efficiency in membranes, as shown by zwitterionic surface layers in nanofiltration systems.^311^ Agricultural residues exhibit a spectrum of these behaviors depending on processing, with lignin-rich raw fibers favoring sorption, while alkali-oxidized fibers expose mesopores and surface charges that support filtration and separation.^312^ Examples such as plantain pseudostem and sugarcane fibers functionalized with TiO_2_, graphene oxide, or stearic acid demonstrate enhanced crude oil absorption and oil–water separation through combined morphological and chemical modification, while variations in fiber cross-section and intrinsic composition further influence performance across sorption, filtration, and separation functions.^312^

## 5 Extraction and processing of natural fibers

Extraction of natural fibers involves separating fibrous materials from plant tissues using biological or mechanical methods. The most common techniques include dew retting, water retting, and mechanical extraction, each offering specific benefits and limitations depending on conditions and fiber type. A brief comparison of these methods is presented in Table 6.

**Table 6:** Extraction techniques for natural fiber extraction

Extraction technique	Description	Duration	Benefits	Drawbacks	Ref.
Dew retting	Cellulose-rich plant materials are uniformly spread on grassy fields to undergo the combined influence of microorganisms, sunlight, air, and dew, which facilitate fiber separation	Typically takes 2–4 weeks, depending on local environmental conditions	Suitable for regions experiencing heavy dew, warm daytime temperatures, and limited water resources	Results in darker-colored fibers of slightly lower quality; agricultural land remains occupied for several weeks, and fibers are often contaminated with soil or fungal growth	313–316
Water retting	Plant stalks or parts rich in cellulose are submerged in natural water sources such as ponds, rivers, or tanks, allowing microbial action to aid in retting under regular supervision	Usually completed in 1–2 weeks, depending on the water's purity and temperature	Cost-effective, environmentally friendly, and simple to manage; produces uniform and high-quality fibers	Requires substantial amounts of clean water and skilled labor; inadequate monitoring can lead to inferior fiber quality	8 and 317–319
Mechanical extraction	Fibers are separated mechanically using equipment such as hammer mills or decorticators that crush plant tissues and break down adhesive substances binding the fibers	Duration varies based on the fiber yield and production scale	Enables the rapid extraction of large quantities of short fibers suitable for industrial use	Involves high operational costs, increased energy consumption, and potential environmental impacts compared with other methods	56 and 320

### 5.1 Chemical extraction method

Chemical extraction is one of the most widely used approaches for separating fibers from plant stems and leaves, particularly when rapid and efficient removal of non-cellulosic materials is desired. Chemical maceration, also referred to as surfactant retting, involves the use of a heated chemical solution to degrade pectin and other binding substances that hold fibers together within plant tissues.^321^ The process facilitates the detachment of the fibrous bundles from woody or cortical material, yielding cleaner and more uniform fibers. The retting solution typically contains chemical agents such as potassium hydroxide (KOH), sulfuric acid (H_2_SO_4_), calcium hypochlorite (Ca(ClO)_2_), sodium carbonate (Na_2_CO_3_), or sodium hydroxide (NaOH).^322^ These chemicals promote hydrolysis of pectic and hemicellulosic substances and, when combined with surface-active agents, help emulsify and remove non-cellulosic components such as lignin, waxes, and pectins through micelle and emulsion-forming mechanisms.^323^

Prior to chemical retting, harvested plant materials may be milled, crushed, or mechanically decorticated to increase the surface area and improve chemical penetration during processing. The treatment conditions, such as temperature, chemical concentration, and duration, are critical in determining fiber quality. Depending on these parameters, retting may last from a few minutes up to 48 hours.^23^ Following chemical retting, the fibers are thoroughly washed with running water, then dried and combed to remove residual chemicals and impurities. This process typically produces high-purity fibers with smooth surfaces, uniform texture, and excellent adhesion properties for subsequent spinning, finishing, or composite reinforcement.^321^ However, the disadvantages of this method include high operational costs, potential fiber damage under excessive chemical exposure, and water pollution resulting from effluent disposal, which have prompted ongoing research into more sustainable alternatives.

Recent advances have focused on optimizing chemical retting to reduce environmental impact and improve fiber quality. Nassar *et al.*^324^ developed a novel multi-step chemical extraction technique consisting of dewaxing, acetylation, and mercerization, with controlled reaction times and optional microwave-assisted heating. This sequential process effectively removes waxes and amorphous hemicellulose, increases fiber crystallinity, and enhances tensile strength, producing cellulose-rich fibers suitable for advanced bio-composite applications. Similarly, Balasubramanian *et al.*^325^ introduced a modified low-concentration alkali treatment for jute fibers using only 2% NaOH, followed by ethanol–acetic acid neutralization. This approach significantly reduces lignin and hemicellulose content while maintaining fiber integrity, resulting in cleaner surfaces and improved interfacial bonding with reduced chemical usage. The method offers an eco-friendlier and cost-effective alternative to traditional high-alkali treatments, supporting sustainable practices in natural fiber processing.

### 5.2 Biological methods

#### 5.2.1 Water retting

Water retting is one of the oldest and most widely used biological extraction methods for separating fibers from plant stalks, particularly bast fibers such as flax, jute, kenaf, hemp, and ramie. In this process, the fiber-bearing plants are submerged in water for a period of 3 to 35 days, during which naturally occurring microorganisms decompose the pectin and hemicellulose that bind the fibers to the woody core and epidermis.^326^ Retting can be performed in running water bodies such as rivers and streams, or in static tanks or ponds, where the water is replaced every few days to maintain bacterial activity and prevent stagnation. The duration of retting depends largely on water temperature and microbial activity, with higher temperatures (around 28–40 °C) accelerating the process and enabling fiber separation within 3–5 days.^323^

The process primarily involves anaerobic bacterial action, where microorganisms such as *Clostridium* species digest the gummy substances holding the fibers together, allowing them to be easily extracted after drying and combing. Water-retted fibers are known for their superior fineness, color, luster, and strength compared to those obtained through mechanical or chemical methods. However, traditional water retting has several drawbacks, including high water consumption, the need for large soaking areas, and environmental pollution caused by the release of organic waste and fermentation gases.^327^ Furthermore, the method requires specialized drying and handling equipment, making it labor-intensive and costly. Due to these environmental and economic challenges, industrial-scale water retting has been banned or restricted in many regions, including much of Europe.^322^

Recent innovations aim to make water retting more sustainable and resource-efficient. A modern technique known as micro-pond retting reduces water usage by up to one-sixth of the traditional process through the use of microbial inoculation and water recycling systems.^328^ This method integrates aquaculture and crop production, utilizing retting residues as nutrient sources while minimizing environmental discharge. Hossain *et al.*^329^ demonstrated that alternative water sources such as tap, pond, and canal water could be effectively used for retting kenaf fibers, producing high-quality fibers with varying cellulose and lignin contents. Their findings suggest that fiber quality can be maintained without relying on large freshwater volumes, offering a more adaptable and sustainable approach to retting. Similarly, Harsányi *et al.*^330^ developed a controlled anaerobic water retting (AWR) process for flax fiber extraction. This system operates in closed bioreactors under strictly anaerobic conditions, eliminating the need for added chemicals or enzymes. The AWR process not only yields high-purity cellulose-rich fibers but also produces valuable by-products such as organic acids (notably acetic and butyric acid), hydrogen-rich gas, and biomethane.

#### 5.2.2 Dew retting

Dew retting is considered the oldest and simplest biological method for separating fibers from plant stems, and it remains one of the most widely used retting techniques globally.^322^ In this process, fiber-yielding crops such as flax, hemp, and jute are harvested and spread evenly on open fields, where natural moisture, temperature, and microbial activity facilitate fiber separation. The process typically takes 3 to 6 weeks, depending on environmental conditions such as humidity, rainfall, and air temperature. These factors influence the growth and metabolic activity of microorganisms, primarily fungi and bacteria, which gradually degrade the pectin and hemicellulose that bind fibers to the plant cortex and woody tissues.^323^

During dew retting, the crops are exposed to alternating wet and dry conditions, promoting the activity of aerobic microorganisms such as *Clostridium*, *Bacillus*, *Pseudomonas*, and *Aspergillus* species. These microbes produce pectinolytic and hemicellulolytic enzymes that break down non-cellulosic components, allowing the fibers to be separated more easily from the xylem. However, the success of dew retting depends heavily on environmental control and careful monitoring. Improper timing can result in over-retting, where excessive microbial action leads to partial degradation of cellulose and loss of fiber strength, or under-retting, where incomplete degradation leaves fibers difficult to extract and process.^331^ Therefore, continuous observation of weather conditions, periodic turning of plants for uniform exposure, and optimization of retting duration are crucial to ensure homogenous retting and maintain fiber quality.

Despite its simplicity and low cost, dew retting has inherent limitations. Its dependence on weather conditions makes it unpredictable and unsuitable for regions with inconsistent temperature or humidity levels. Moreover, fibers obtained through dew retting are often darker in color and exhibit lower fineness and strength compared to those produced by water or chemical retting, primarily due to prolonged contact with soil and partial degradation of the fiber surface.^327^ Nevertheless, the method remains popular among small-scale and sustainable fiber producers because it requires minimal energy, water, and chemical input, aligning with eco-friendly agricultural practices.

Recent advancements aim to improve control and efficiency in dew retting by integrating microbial monitoring and biotechnology tools. Orm *et al.*^332^ introduced a microbial enzyme-linked monitoring framework for hemp dew retting, employing high-throughput sequencing to correlate microbial taxa and enzymatic profiles with retting stages. This study identified *Pseudomonas*, *Sphingomonas*, and *Cladosporium* as key microbial indicators of retting progress, enabling better prediction and regulation of retting outcomes. Such biotechnological approaches enhance understanding of microbial succession and enzymatic activity in field retting environments, paving the way for more consistent fiber quality and shorter processing times.

#### 5.2.3 Enzymatic retting

Enzymatic retting is a biotechnologically advanced fiber extraction method that utilizes specific enzymes to degrade the pectin and hemicellulose binding the fibers to plant tissues, thereby facilitating their separation. Unlike traditional retting methods that rely on uncontrolled microbial activity or harsh chemicals, enzymatic retting offers greater precision, uniformity, and fiber quality. The process begins soon after harvesting, when plants are lightly crushed or crimped using mechanical rollers to break the outer epidermis and increase enzyme penetration into the bast tissue.^333^ The prepared plant material is then incubated in a controlled tank environment containing a pectinolytic enzyme solution, often supplemented with xylanase or mannanase enzymes, at an optimized temperature and pH that support maximum enzymatic activity.^334^ The retting duration typically ranges between 2 and 24 hours, depending on the enzyme concentration, plant type, and temperature conditions.

The enzymes used in this process, such as pectinase, polygalacturonase, xylanase, and mannanase, specifically target pectin and hemicellulose, degrading the non-cellulosic cementing materials while preserving the cellulose microfibrils that impart strength to the fibers. As a result, enzymatic retting yields high-quality fibers characterized by smooth surfaces, high crystallinity, and superior mechanical performance. Additionally, the controlled enzymatic action minimizes fiber damage and color alteration compared to chemical or water retting methods. However, the main drawbacks of enzymatic retting are its high operational cost, attributed to enzyme production and purification, and the need for wastewater treatment and specialized equipment, which limit its industrial scalability.^335^

Recent studies have explored microbial consortia and enzyme-based bioaugmentation systems to make enzymatic retting more efficient and cost-effective. Ventorino *et al.*^336^ developed a microbial consortia-based retting approach for hemp fibers, employing selected strains of *Bacillus*, *Paenibacillus*, and *Pseudomonas* that exhibit synergistic enzymatic activity. The consortium displayed high pectinolytic activity while maintaining cellulose integrity, achieving effective fiber separation within five days and producing fibers with improved fineness and tensile properties. Similarly, Huang *et al.*^337^ introduced a fungal consortium-based bioaugmentation retting system for sisal fibers, consisting of *Aspergillus micronesiensis*, *Penicillium citrinum*, and *Cladosporium* species. This combination generated high levels of pectinase, xylanase, and mannanase, enhancing degumming efficiency and cellulose purity without causing cellulase-induced fiber degradation. The approach not only improved retting speed but also produced fibers with enhanced cleanliness, whiteness, and bonding characteristics suitable for textile and composite applications.

### 5.3 Hybrid extraction methods

#### 5.3.1 Microwave-assisted retting

Microwave-assisted retting is a modern and energy-efficient technique developed to accelerate the separation of fibers from lignocellulosic plant materials through the use of microwave radiation. Unlike conventional retting methods that rely on slow microbial or chemical degradation, this approach utilizes dielectric heating, in which polar molecules—primarily water—absorb microwave energy and convert it into heat *via* molecular vibration and friction. The rapid and uniform heating generated by microwaves causes disruption of the middle lamella, which weakens the pectic and hemicellulosic bonds binding the fiber bundles to surrounding tissues.^338^ This process enables quick loosening of fiber bundles from the plant matrix, dramatically reducing retting time compared to traditional methods such as dew or water retting.

The primary advantage of microwave-assisted retting lies in its ability to provide precise control over process parameters, including temperature, exposure time, and moisture content. Controlled microwave energy ensures uniform heating throughout the plant material, resulting in consistent fiber quality and color while minimizing fiber damage. The process also significantly reduces microbial contamination, water consumption, and chemical usage, making it a cleaner and more sustainable alternative to conventional retting methods.^339^ Furthermore, microwave treatment can be integrated with mild chemical or enzymatic pretreatments, enhancing the degradation of pectins and hemicelluloses while preserving cellulose integrity. The combination of microwaves with alkaline or enzymatic solutions has been shown to produce fibers with smoother surfaces, improved purity, and enhanced mechanical properties, suitable for high-performance textile and composite applications.

In addition to its environmental advantages, microwave-assisted retting effectively eliminates issues such as odor emission, effluent generation, and prolonged fermentation, which are common drawbacks of biological and water-based retting techniques. However, the technology also presents certain limitations. The initial capital investment for industrial-scale microwave equipment is relatively high, and the process requires careful optimization to avoid localized overheating, which can lead to thermal degradation of cellulose and reduced fiber strength.^340^ Factors such as plant moisture content, fiber density, and radiation power must be precisely controlled to achieve optimal results. Despite these challenges, microwave-assisted retting is emerging as a promising, eco-friendly, and time-efficient fiber extraction technology. Its potential to combine speed, sustainability, and product quality makes it a valuable innovation in the modernization of natural fiber processing, offering an advanced alternative to traditional retting practices for the textile industry.

#### 5.3.2 Duralin process

The Duralin process, developed by Ceres B.V., is a thermo-chemical method designed to enhance the dimensional stability and environmental resistance of bast fibers such as flax and hemp.^341^ The process consists of three main stages: hydrothermolysis, drying, and curing.^342^ In the first stage, the plant stems are treated with steam in an autoclave at 160 °C for 30 minutes, which softens the lignocellulosic structure and partially breaks down lignin and hemicellulose. The stems are then oven-dried to remove moisture, followed by curing at 150 °C for 2 hours. During curing, the degraded lignin and hemicellulose components form low-molecular-weight aldehydes and phenolic compounds, which polymerize into a natural resin that binds the cellulose microfibrils together, improving water resistance and strength.^343^

After treatment, the fibers are extracted by crushing, decortication, or scutching, producing clean, high-quality bast fibers with enhanced physical and surface properties suitable for composite and technical textile applications.^208^ The Duralin process eliminates the need for conventional retting and yields strong, dimensionally stable, and hydrophobic fibers, making it a sustainable and efficient alternative for high-performance natural fiber production.^341^

### 5.4 Emerging methods

Modern research in natural fiber processing has led to the development of several innovative extraction techniques that aim to improve efficiency, reduce environmental impact, and enhance fiber quality. Among these, steam explosion, ultrasound retting, and stand retting are notable for their effectiveness in fiber separation, shortened processing times, and potential for industrial scalability.

The steam explosion method (STEX) is a thermo-mechanical fiber extraction process that utilizes high-pressure steam, temperature, and rapid decompression to separate fibers from lignocellulosic plant materials.^321^ In this technique, the dried plant stems are exposed to high-pressure saturated steam, which penetrates the plant tissues and softens the lignin and hemicellulose components. The process is followed by a sudden pressure release, generating intense thermomechanical shear forces that rupture the middle lamella, the binding layer between fibers, leading to efficient fiber separation.^322^ The resulting fibers exhibit fine texture, smooth surfaces, and mechanical properties comparable to cotton, making this method highly suitable for textile-grade natural fibers. In addition to producing high-quality fibers, steam explosion requires less chemical input and water, thus offering an eco-friendlier and rapid alternative to traditional retting techniques.

Another innovative technique, ultrasound retting, combines acoustic cavitation and mild chemical treatment to accelerate fiber extraction. In this process, cleaned and crushed plant stems are submerged in a hot water bath (around 70 °C for 24 h) containing low concentrations of alkali and surfactants, and then exposed to high-intensity ultrasound (1 kW, 40 kHz).^344^ The ultrasonic vibrations generate microscopic cavitation bubbles that collapse near the fiber surfaces, producing localized shock waves and micro-jets that break down the pectin-rich matrix binding the fibers. This results in faster separation, smoother fiber surfaces, and improved uniformity compared to conventional water retting. While the method significantly reduces retting time and enhances fiber fineness, cleanliness, and mechanical strength, large-scale industrial implementation remains limited due to high equipment cost and energy requirements.^345^ Nevertheless, ultrasound-assisted retting represents a promising green technology for next-generation fiber extraction.

The stand retting method is a modified version of dew retting, developed to address some of its limitations. In one variation, the fiber crops are treated in the field before harvest with glyphosate (*N*-phosphonomethyl glycine), a desiccant that facilitates fiber separation by weakening the plant tissues.^322^ This approach yields fibers with improved mechanical properties compared to traditional dew retting.^346^ Another variation involves thermal treatment, where the lower sections of standing plants are heated to about 100 °C and subsequently dried for several days.^323^ This method reduces dependency on weather conditions and accelerates retting. Although stand retting minimizes risks of fiber damage from rain or prolonged field exposure, it incurs higher operational costs due to the need for controlled heating systems.^347^ Moreover, the use of glyphosate-based chemicals raises environmental and health concerns, including soil toxicity and adverse effects on microbial and aquatic ecosystems,^348^ making its use undesirable in sustainable fiber production.

## 6 Modification of natural fibers

### 6.1 Alkaline treatment

**Fig. 14:**
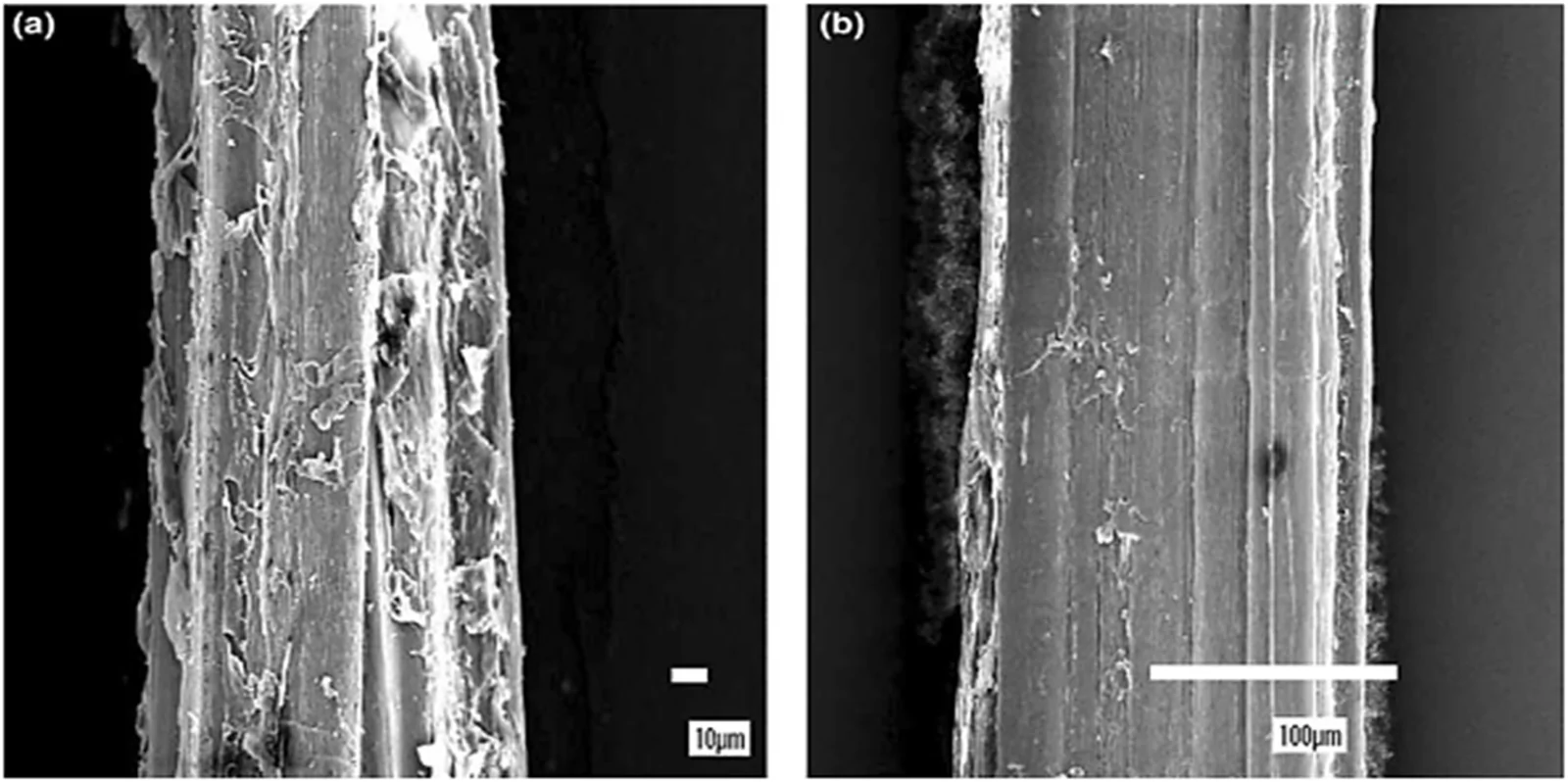
SEM micrographs of (a) untreated hemp fibre and (b) 6% NaOH treated hemp fibre. This figure has been reproduced from ref. 354 with permission from Elsevier, copyright 2003.

The alkaline treatment (also known as alkali or mercerization treatment) is one of the simplest, most economical, and effective methods for improving the adhesion and interfacial bonding between natural fibers and polymer matrices.^349^ In this process, the fibers are treated with an aqueous sodium hydroxide (NaOH) solution, which modifies the cellulosic molecular structure and enhances mechanical interlocking with the matrix. The treatment increases fiber fragmentation and disaggregation, altering the orientation of crystalline cellulose and creating amorphous regions where water molecules penetrate and separate cellulose microfibrils.^350^ The alkali reacts with hydroxyl (–OH) groups in cellulose, forming fiber-O–Na bonds and reducing the number of hydrophilic groups, thereby increasing moisture resistance.^351^ This process also removes hemicellulose, lignin, pectin, wax, and oil, resulting in a cleaner and more uniform surface, which improves stress transfer between fiber and matrix.^352^ The treatment decreases fiber diameter, increases the effective surface area and aspect ratio, and improves adhesion strength, provided that the alkali concentration is optimized; excessively high concentrations can cause over-delignification and fiber damage. Brígida *et al.*^353^ found that alkaline treatment is the most efficient technique for exposing cellulose in natural fsibers and that it enhances the thermal stability of green coconut fibers while maintaining their hydrophilic character. Fig. 14 shows SEM micrographs of longitudinal views of untreated and 6% alkaline-treated kenaf fibers, where surface impurities are visibly removed, and smoother, cleaner fiber surfaces are observed.

### 6.2 Silane treatment

The silane treatment method is a widely used chemical surface modification technique designed to improve the adhesion between natural fibers and polymer matrices by forming stable covalent bonds at the interface. In this approach, silane coupling agents are applied to the fiber surface, where they coat the micro-pores and irregularities, creating a molecular bridge between the hydrophilic fiber and the hydrophobic matrix.^355^ During the initial stage, silanols are generated through the hydrolysis of the alkoxy groups in the silane compound in the presence of moisture. These silanols then react with the hydroxyl (–OH) groups of cellulose on the fiber surface, forming Si–O-cellulose bonds, while the other reactive end of the silane molecule condenses with the functional groups of the matrix, producing a siloxane (Si–O–Si) bridge.^356^ This condensation reaction establishes molecular continuity across the interface, resulting in improved chemical compatibility and load transfer between the two phases. The hydrophobic hydrocarbon chains of silane further reduce fiber swelling and moisture absorption, enhancing the dimensional stability of the composite.^352^ Fibers treated with silane show superior tensile strength and interfacial adhesion compared to those modified only by alkali treatment.^357^

### 6.3 Acetylation treatment

The acetylation treatment is a chemical modification process that enhances the dimensional stability, moisture resistance, and interfacial adhesion of natural fibers by introducing acetyl groups (–COCH_3_) into their cellular structure.^358^ In this method, the fibers are first soaked in acetic acid and then treated with acetic anhydride in the presence of an acid catalyst for 1–3 hours at elevated temperatures, as acetic acid and acetic anhydride alone cannot react effectively with the fiber surface.^13^ During treatment, an esterification reaction occurs between the hydroxyl (–OH) groups of the cellulose and the carboxylic or anhydride groups, resulting in the formation of ester linkages within the fiber structure.^359^ This substitution of hydroxyl groups by acetyl groups reduces the number of hydrophilic sites, thereby lowering the fiber's ability to absorb moisture and swell.

**Fig. 15:**
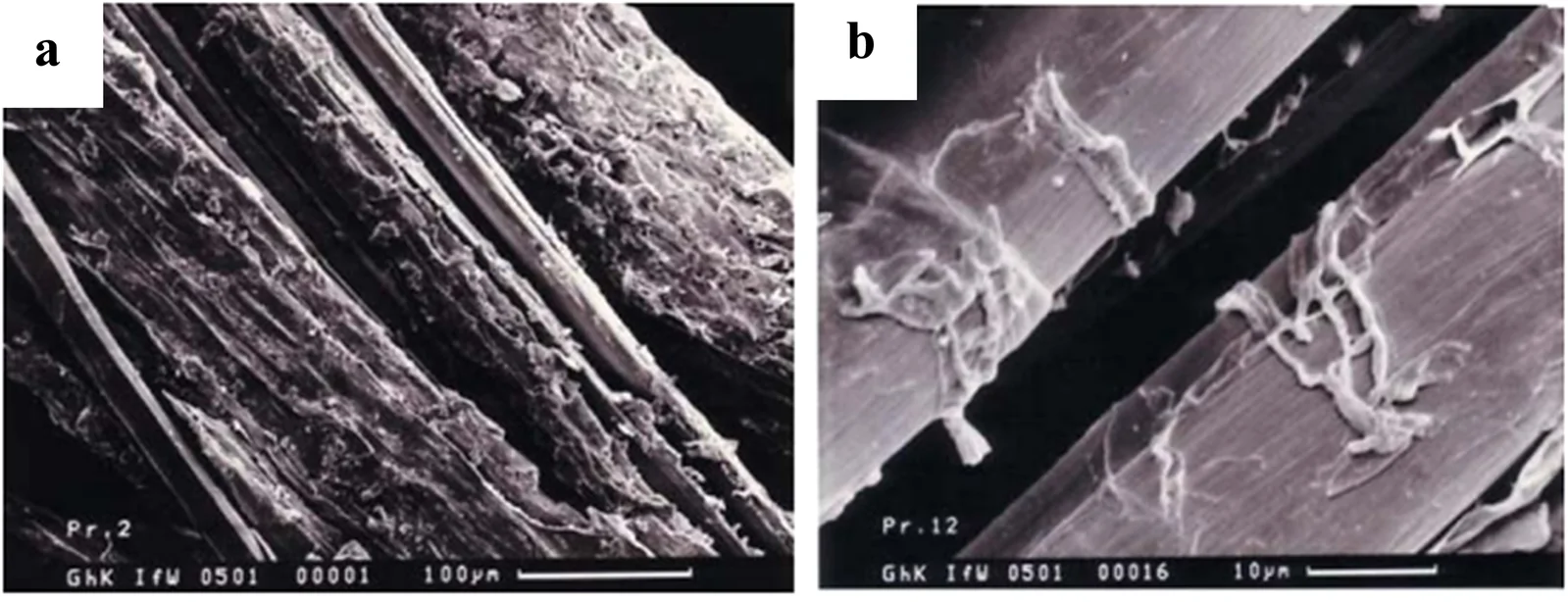
SEM micrographs showing the surface morphology of (a) untreated and (b) 18% acetylation-treated flax fibers. This figure has been reproduced from ref. 362 with permission from BME-PT, copyright 2021.

The acetylation process also removes surface waxes and cuticular layers, producing a smoother and more uniform fiber surface with improved compatibility with hydrophobic polymer matrices. According to Tserki *et al.*,^360^ acetylation of flax and hemp fibers significantly decreases moisture absorption, eliminates non-crystalline constituents, and improves stress transfer efficiency due to better interfacial bonding. Zafeiropoulos *et al.*^361^ further reported that acetylation alters both the bulk and surface properties of flax fibers, leading to enhanced dimensional stability and mechanical behavior in composite materials. Fig. 15 presents SEM micrographs of surface views of untreated and 18% acetylation-treated flax fibers, where the treated fibers exhibit a cleaner and smoother morphology compared to untreated samples.

### 6.4 Peroxide treatment

The peroxide treatment is a chemical surface modification technique that enhances the adhesion, thermal stability, and moisture resistance of natural fibers by introducing reactive radicals onto their surfaces. In this method, peroxide-induced grafting, typically involving compounds such as hydrogen peroxide or organic peroxides, is used to anchor polyethylene or other coupling agents onto the fiber surface.^363^ When activated, the peroxide molecules decompose to form free radicals, which react with the hydroxyl (–OH) groups present in the cellulose structure of the fibers and with the functional groups of the polymer matrix. This reaction promotes strong covalent bonding at the fiber–matrix interface, resulting in improved stress transfer and better compatibility between the hydrophilic natural fiber and the hydrophobic polymer matrix.^131^

**Fig. 16:**
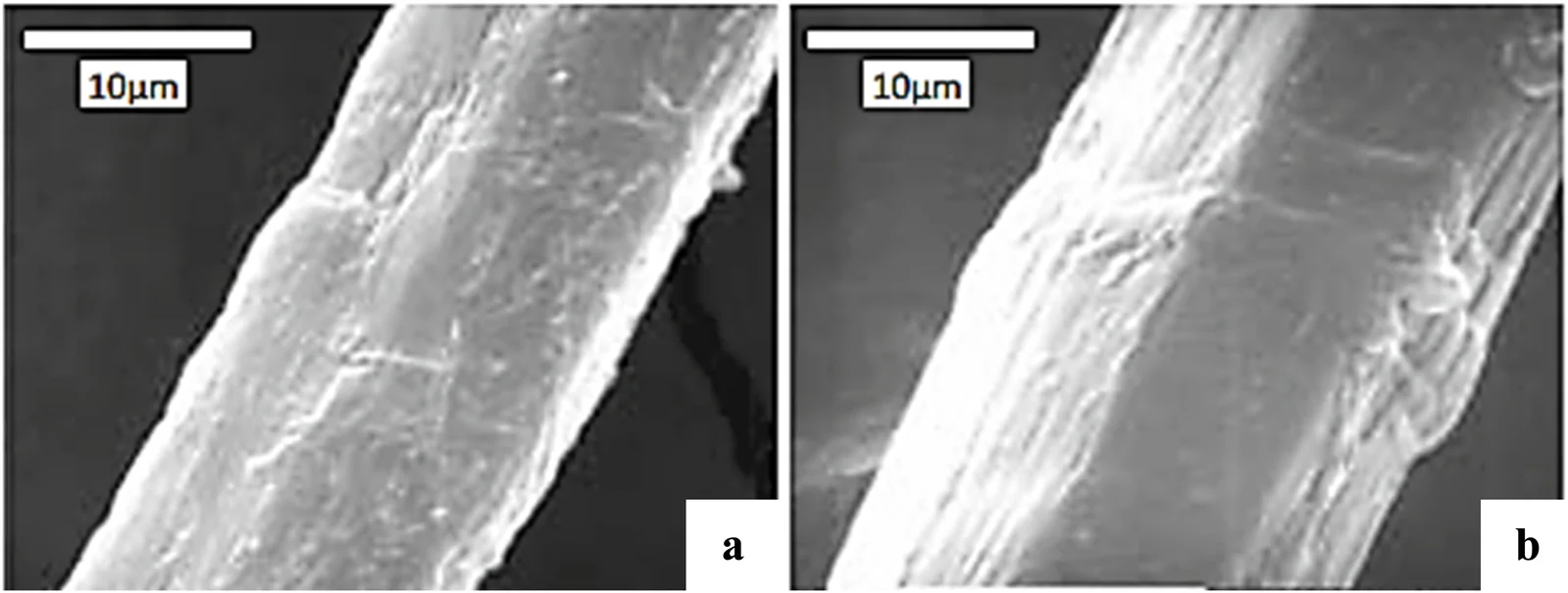
SEM images depicting surface modifications of flax fibers: (a) untreated and (b) after peroxide treatment. This figure has been reproduced from ref. 365 with permission from SAGE Publications, copyright 2026.

Peroxide treatment also leads to the reduction of hydroxyl groups, thereby decreasing the moisture absorption capacity of the fibers and enhancing their dimensional and thermal stability.^364^ The resulting fibers exhibit smoother surfaces and reduced porosity, which contribute to improved mechanical performance in composite applications. Fig. 16 shows SEM micrographs of surface views of untreated and peroxide-treated flax fibers, clearly illustrating the removal of surface impurities and the formation of a cleaner, more uniform surface morphology after treatment.

### 6.5 Corona treatment

**Fig. 17:**
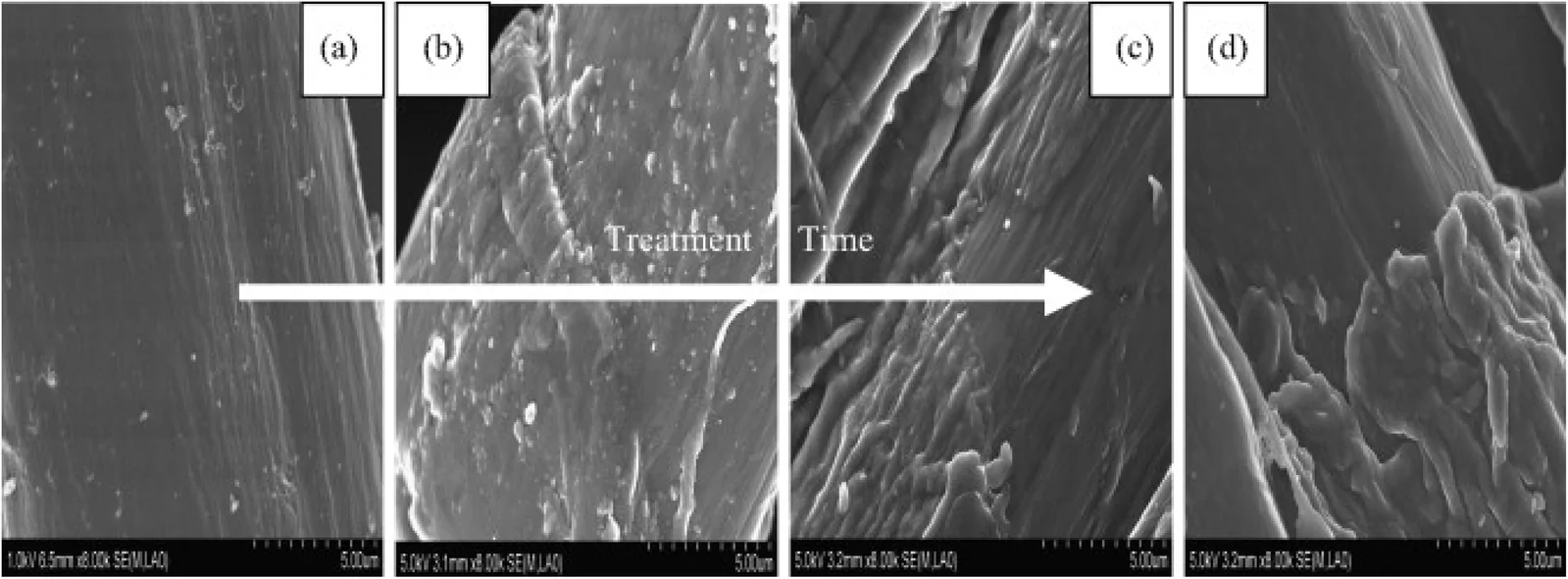
SEM images of corona-treated hemp fiber (a) untreated, (b) after 15 min, (c) after 30 min, (d) after 40 min. This figure has been reproduced from ref. 368 with permission from Elsevier B.V., copyright 2009.

Corona treatment is a physical surface modification method that increases the surface energy of natural fibers through oxidation, improving compatibility between hydrophilic fibers and hydrophobic matrices.^366^ The process involves two aluminum electrodes separated by a quartz dielectric spacer, where electrical discharge passes through an air gap to treat the fiber surface.^367^ Since fibers are placed on the electrode surface, mainly one side is exposed, though in composites, the entire lateral surface contributes to reinforcement.^368^ SEM images, as presented in Fig. 17, show that longer treatment times produce rougher fiber surfaces, enhancing mechanical interlocking between fiber and matrix. The treatment activates surface sites along polymer chains, allowing oxidation and etching, leading to up to 30% improvement in Young's modulus and tensile strength of hemp and *Miscanthus*–polypropylene composites, while composites with polylactic acid show around 20% improvement.^369^ Although corona treatment modifies fiber surfaces effectively, potential fiber damage and the long-term stability of the interfacial bonding require further investigation.

## 7 Environmental impact and sustainability analysis

**Fig. 18:**
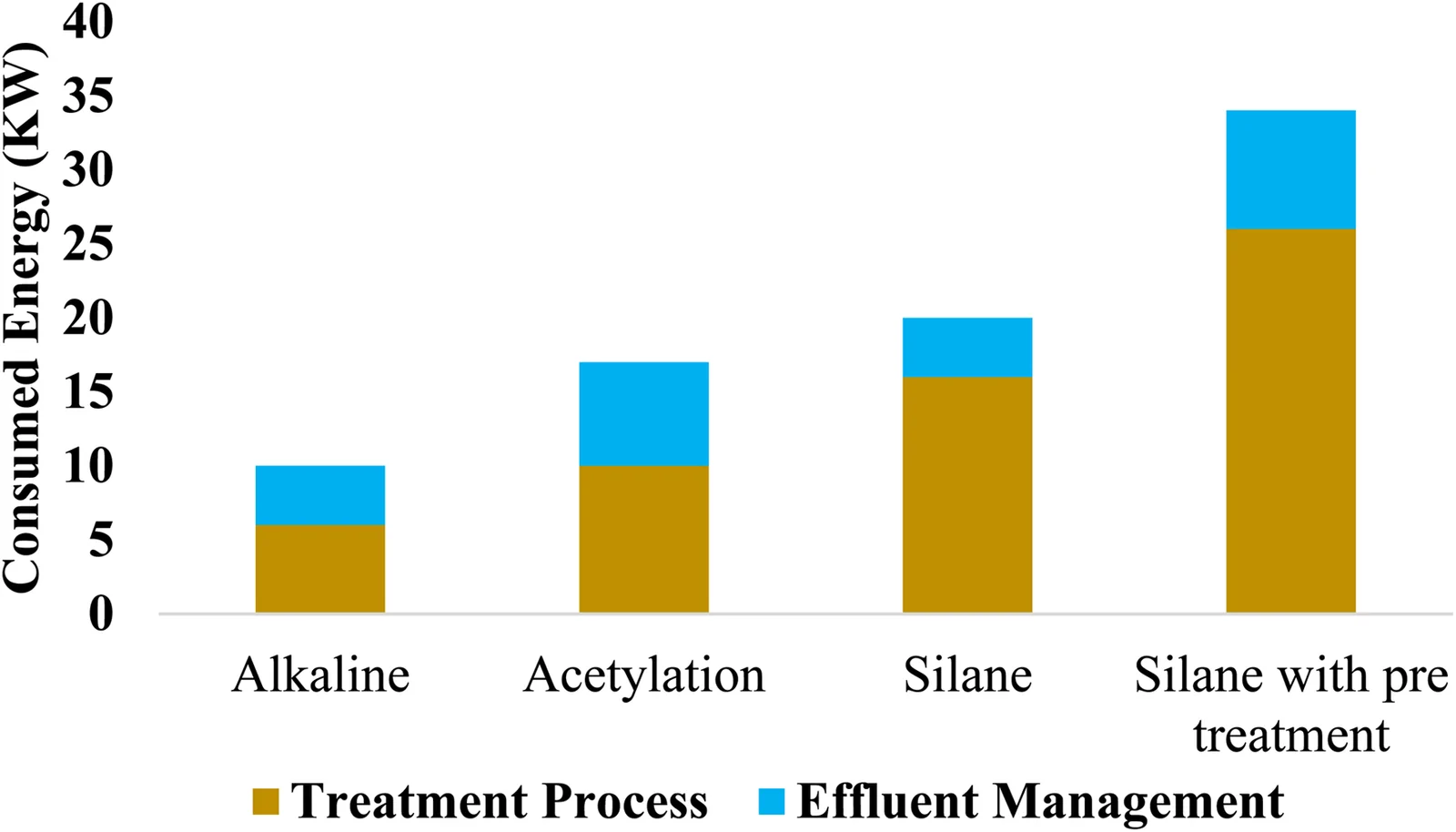
Energy consumption for different chemical treatments per unit of natural fiber.

Energy consumption during natural fiber processing and modification is a key contributor to environmental impact and depends strongly on treatment type, duration, and effluent generation. To evaluate energy demand per unit of treated fiber, the fiber source and energy source were kept constant. A 1 hour oven drying process at 120 °C was assumed to consume 1.1 kW, while 1 hour of laboratory mixing using a standard mixer required 216 W.^370^ Chemical treatments such as silane and acetylation generally involve pre-treatment steps, with chemical concentrations ranging from 1% to 5%. Alkaline and acetylation treatments typically require about 2 hours, whereas silane treatment extends up to 12 hours due to slow hydrolysis and condensation reactions governing silane coupling to fiber surfaces. Maleated treatment occurs during composite manufacturing and therefore does not require separate processing time or effluent handling. As shown in Fig. 18, total energy consumption includes both process energy from mixing, washing, and drying and energy associated with effluent management, which increases with effluent volume.^370^ Alkaline treatment, involving 2 hours of processing and 6 hours of drying at 120 °C, consumed approximately 10 kW, while acetylation treatment required about 17.5 kW due to multiple processing and drying steps. Silane treatment exhibited higher energy demand because of prolonged mixing at 60 °C, and this increased further when pre-treatment was applied. When comparing pre-treated acetylation and silane treatments, acetylation showed higher effluent-related energy consumption, whereas silane treatment required higher process energy due to extended treatment duration.^370^

The higher energy demand associated with chemical modification routes is directly linked to the underlying reaction mechanisms responsible for surface functionalization and by-product formation:

Alkaline treatment (mercerization): Cell–OH + NaOH → Cell–O^−^ Na^+^ + H_2_O

Acetylation: Cell–OH + (CH_3_CO)_2_O → Cell–OCOCH_3_ + CH_3_COOH

Silane treatment (hydrolysis and condensation): R–Si(OR′)_3_ + 3H_2_O → R–Si(OH)_3_ + 3R′OH Cell–OH + R–Si(OH)_3_ → Cell–O–Si–R + H_2_O

These reactions explain the increased energy demand observed in Fig. 18, as alkaline and acetylation treatments generate significant aqueous effluents requiring repeated washing and drying, while silane treatments demand prolonged thermal activation to complete hydrolysis and condensation at the fiber surface. Maleated coupling, in contrast, proceeds through *in situ* radical grafting during melt processing and therefore avoids additional solvent use and post-treatment drying, resulting in lower net energy input per unit fiber.

Beyond processing energy, natural fiber production and utilization are closely linked to socio-economic sustainability, particularly in fiber-producing regions where these materials support livelihoods and local industries. Natural fibers offer low production cost, wide availability, low density, favorable mechanical performance, thermal and acoustic insulation, and reduced health risks during processing, supporting their continued use in textile and composite applications.^371^ In countries such as India, Bangladesh, and Kenya, cultivation and processing of cotton, jute, hemp, sisal, and related fibers provide employment for millions of rural workers and contribute significantly to export-oriented economies.^372^ In Indonesia, initiatives such as the Ramie Consortium (KORI) and the Natural Fiber Council (DSI) promote integrated systems covering cultivation, fiber extraction, spinning, and weaving of fibers including abaca, kenaf, bamboo, pineapple, sisal, cotton, and ramie, reducing dependence on imported cotton and strengthening domestic production chains.^373^ These efforts are complemented by the increasing utilization of agricultural residues such as pineapple leaves, banana stems, rice straw, and palm waste as alternative fiber sources within circular production approaches.^374^ Community-based extraction units and small-scale enterprises across Southeast Asia employ traditional retting and decortication methods to produce fibers for handicrafts, apparel, and home textiles, providing additional income for farmers despite low yields, such as 2.5 kg of pineapple fiber from 100 kg of fresh leaves.^375^

At the industrial level, these socio-economic benefits intersect with environmental considerations as natural fibers are increasingly incorporated into bio-based textiles and fiber–reinforced composites for automotive interiors, furnishings, and protective applications, offering lightweight and cost-effective alternatives to synthetic fibers.^376^ Government incentives and national research programs further support these developments by encouraging collaboration between academia, industry, and farming communities and promoting eco-packaging and biotextiles derived from agricultural residues.^377^ The adoption of circular economy practices, including reuse, recycling, and upcycling of fiber waste, improves resource efficiency and supports local employment while reducing material losses across the value chain.^378^

Despite these advantages, the textile and fashion industries continue to exert substantial environmental pressure due to intensive resource use, energy consumption, and waste generation. The production of natural fibers such as wool, cotton, and leather contributes to water pollution, land degradation, and greenhouse gas emissions, particularly methane, which has a significantly higher global warming potential than carbon dioxide.^379^ Methane emissions largely originate from livestock associated with wool production, and their reduction provides a rapid pathway for climate mitigation due to methane's short atmospheric lifetime. Strategies such as increasing the use of recycled wool, transitioning from animal-based to next-generation leather, adopting renewable electricity, electrifying processing stages, and using low-carbon transport fuels can substantially reduce emissions. A complete shift from virgin to recycled wool combined with an 80% transition from animal-based to next-generation leather could nearly achieve a 30% methane reduction target by 2030.^380^ Organizations such as Collective Fashion Justice support brands in quantifying methane footprints and identifying reduction pathways.

## 8 Challenges and opportunities

Although natural fibers are widely recognized for their biodegradability, renewability, and lower environmental footprint compared to synthetic alternatives,^381^ several challenges still hinder their large-scale adoption in the fashion industry. One of the key challenges lies in their dependency on agricultural and climatic conditions, which can cause fluctuations in fiber quality and yield. The cultivation of certain natural fibers, such as cotton, demands significant water and land resources and often involves the use of pesticides and fertilizers that contribute to soil degradation and water pollution.^382^ Similarly, animal-based fibers like wool, leather, and silk, though renewable, can generate high carbon emissions and raise ethical concerns regarding animal welfare.^383^ Furthermore, natural fibers may lack some of the functional advantages of synthetics, such as elasticity, moisture resistance, and extended durability, making them less suitable for performance wear.^384^ Processing methods for natural fibers also tend to be less efficient, and their mechanical properties can vary depending on factors such as fiber type, growth conditions, and extraction techniques.^385^

Despite these challenges, natural fibers offer substantial opportunities for advancing sustainability and innovation in the textile and fashion sectors. Their biodegradability and renewability make them ideal for developing circular and eco-friendly production models that minimize environmental pollution.^386^ The use of locally sourced raw materials such as hemp, flax, jute, and kenaf not only reduces transportation-related emissions but also supports regional economies and promotes soil regeneration through carbon sequestration and heavy metal absorption. Additionally, advancements in fiber processing technologies, such as enzymatic treatments, blending with elastomers, and eco-friendly coatings, are improving the mechanical and comfort properties of natural fibers, allowing them to compete more effectively with synthetic materials.^387^ As sustainability becomes a core strategic priority for modern businesses,^388^ the fashion industry has an opportunity to leverage natural fibers not only to reduce its ecological footprint but also to align with consumer demand for ethical, durable, and environmentally responsible products. With continued innovation, education, and policy support, natural fibers can play a pivotal role in transforming fashion into a more sustainable and resilient global industry.

## 9 Conclusion

This review demonstrates that natural fibers should not be viewed as a single sustainable solution but as a diverse group of materials whose performance and environmental relevance depend on source-specific structure, chemistry, and processing routes. Plant-based fibers such as cotton, jute, hemp, flax, bamboo, and kapok exhibit wide variation in cellulose crystallinity, lignin content, and lumen morphology, which governs their mechanical strength, moisture response, and thermal behavior. Animal-derived fibers, including wool, silk, feather fibers, and human hair, introduce protein-based chemistries where keratin and fibroin structures impart elasticity, insulation, and toughness but impose limitations related to thermal stability and processing energy. The comparative discussion with mineral fibers highlights how inorganic structures offer superior thermal resistance while raising critical health and sustainability constraints, reinforcing the need for application-driven fiber selection rather than generalized sustainability claims.

The analysis further shows that chemical modification is essential for improving compatibility and performance but represents a key trade-off between functional gains and environmental burden. Alkali, acetylation, silane, and maleated treatments operate through distinct chemical pathways that alter fiber surface chemistry and interfacial behavior, yet they differ substantially in energy demand, treatment duration, and effluent generation. The energy assessment presented indicates that prolonged treatments, particularly silane-based systems, contribute disproportionately to total environmental impact despite their effectiveness, underscoring the need to evaluate modification strategies beyond mechanical performance alone.

From a sustainability perspective, the environmental benefits of natural fibers are closely tied to regional availability, agricultural practices, and the use of residues rather than dedicated crops. The utilization of pineapple leaves, banana stems, rice straw, and palm waste offers clear advantages by reducing waste streams and supporting rural economies, but these benefits can be diminished by energy-intensive processing or inefficient effluent management. Animal-based fibers further illustrate the complexity of sustainability assessment, where durability and insulation performance must be balanced against emissions and land-use impacts.

Overall, this review integrates fiber structure, chemical modification, processing energy, and environmental impact into a unified framework, identifying moisture sensitivity, interfacial incompatibility, treatment-related energy costs, and supply variability as key constraints. Addressing these limitations through lower-energy chemical strategies, application-specific fiber selection, and improved processing efficiency is essential for translating the sustainability potential of natural fibers into scalable textile and composite applications.

## Author contributions

Sayam: conceptualization, methodology, resources, software, supervision, validation, visualization, writing – original draft preparation, and writing – review & editing.

## Conflicts of interest

No conflicts of interest.

## Data Availability

This review does not contain any original data. All the data were written in the manuscript.
